# Thiol‐Methylsulfone Crosslinked Hydrogels for Cell Encapsulation: Molecular Scale Modulation of Physiochemical Properties

**DOI:** 10.1002/mabi.202500627

**Published:** 2026-02-24

**Authors:** Hafiz Syed Usama Bin Farrukh, Aleeza Farrukh, Syuzanna Hambardzumyan, Therese Steudter, Samuel Pearson, Aránzazu del Campo

**Affiliations:** ^1^ INM – Leibniz Institute For New Materials Saarbrücken Germany; ^2^ Chemistry Department Saarland University Saarbrücken Germany

**Keywords:** 3D cell encapsulation, aryl methylsulfone, crosslinking kinetics, thiol crosslinking

## Abstract

Hydrogels mimicking the mechanical and biochemical features of the cellular microenvironment allow cell encapsulation and facilitate in vitro 3D culture. In addition to biocompatibility and reactivity in physiological conditions, a key criterion for crosslinking chemistry is appropriate gelation kinetics to allow mixing and homogeneous distribution of cells with the hydrogel precursors. We have previously presented aryl methylsulfone/thiol (MS/SH) reaction as a thiol‐reactive cross‐linking system for cell encapsulation in star polyethylene glycol (PEG4) hydrogels with a gelation kinetics in minutes time scale. Remaining experimental challenges for this system are a finer modulation of gelation kinetics and streamlining the synthesis of the prepolymer. Here we present the possibility to tune the gelation kinetics by introducing an electron‐withdrawing substituent at *p‐*position of the aryl MS ring. This variant also presents synthetic advantages. We study the influence of the *p*‐substituent on the physicochemical properties of MS/SH crosslinked hydrogels, and their performance for cell encapsulation. We compare these properties with the PEG‐MS variant containing an electron‐donating linker. The new star poly(ethylene glycol)‐4‐(5‐(methylsulfonyl)‐1*H*‐tetrazol‐1‐yl)benzamide (PEG4‐CONH‐TzMS) shows superior properties as cell encapsulating hydrogel in terms of ease of mixing polymer precursors, faster gelation, homogenous cell distribution and high enzymatic stability.

## Graphical abstract

We have synthesized a new variant of tetrazole methylsulfone (TzMS) precursor as a thiol‐reactive cross‐linking system for homogenous cell encapsulation in star poly(ethylene glycol) with gelation kinetics in minutes time scale. Our strategy entailed introduction of an electron‐withdrawing substituent at p‐position of the TzMS ring to fine tune the reaction rates and streamline the synthesis of the prepolymer.

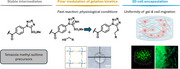

## 1 Introduction

Progress in 3D culture technologies and cell therapies depends on the ability to encapsulate cells in supportive microenvironments for cellular proliferation and self‐organization [1]. Cells encapsulated in natural or synthetic hydrogels are used as scaffolds for tissue engineering in vitro and in vivo [2, 3]. Encapsulated hydrogels are temporal extracellular matrix substituents in these application scenarios [4]. Encapsulation requires hydrogels that can crosslink at physiological conditions (neutral pH, 37°C, aqueous) within a gelation time ideally in the range of ≈60–90 sec. Shorter gelation times do not allow sufficient time for mixing hydrogel precursors and cells, and longer gelation times compromise a homogeneous distribution of cells within the hydrogel due to cell sedimentation [2, 5, 6, 7].

Thiol mediated reactions are attractive for crosslinking hydrogels for cell encapsulation due to several advantageous properties [7, 8, 9]. Thiols exhibit high nucleophilicity at physiological conditions and allow biocompatible substitution and addition reactions with electron‐deficient groups [10]. Thiol nucleophilicity at physiological pH is higher than that of amines or hydroxy groups, enabling thiol reaction with electrophiles to proceed with high selectivity [11]. The reaction kinetics of thiol reactions can be adjusted by pH within the biocompatible range of 6–8 [12, 13]. Functionalization of the hydrogel network with bioactive ligands can be easily done using thiolated ligands [14]. Other advantages include the high stability of resultant thioether bonds and the commercial availability of different thiolated polymeric backbones and crosslinkers.

Different thiol‐X reactions (e.g., thiol‐Michael addition, thiol‐ene radical polymerization, thiol‐halogen ligation, thiol‐epoxy opening, split luciferin ligation, and disulfide bond formation) have been employed for hydrogel crosslinking [15, 16, 17]. Among them, the thiol‐Michael addition is popular for cell encapsulation hydrogels. It involves the reaction of thiols with electron‐deficient alkenes (e.g., vinyl sulfones, VS or maleimide, Mal) and progresses efficiently at mild conditions (i.e., neutral pH, 37°C) to form thioether covalent bonds without the need for light or thermal activation (Table 1) [18, 19]. However, the thiol‐Mal reaction is very fast (reaction completed within a few seconds) at polymer compositions and reaction conditions used for cell encapsulation and can lead to inhomogeneous cell distribution throughout the hydrogel, while the thiol‐VS reaction is slow (several minutes to hours) and can lead to inhomogeneous cell distribution due to the sedimentation of cells during the crosslinking time (Table 1) [20, 21].

**TABLE 1:** Comparison of the reaction rate of thiols and the gelation time observed when these reactions were used for crosslinking star‐PEG functionalized prepolymers.

Reactive groups		Reaction mechanism with thiols	Reaction rate with small molecules^a^ *k2* [M^−1^ s^−1^]	Gelation time of prepolymer^b^ (pH 7)	Cell distribution in 3D hydrogels
Thiol‐Michael addition	VS	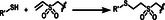	0.08–1 [20]	88 min [24]	Sedimented at the bottom of the gel [24].
	Mal	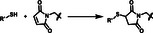	734 [21]	2–3 s [24]	Agglomerated at the upper part of the gel [24].
Thiol‐Methyl sulfone	OxMS	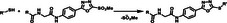	451 ± 46 [25]	12 s [24]	Homogeneously distributed in the gel [22, 24].
	BtMS	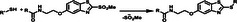	0.027 [25]	23 min [22, 24]	
	*p*‐HO‐TzMS	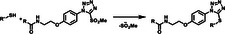	6.03 ± 0.55 [20]	7.1 min [22, 24]	
	*p*‐COOH‐TzMS	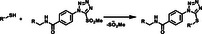	11.2 ± 0.46 [25]	≈60 s	

^a^ Reaction rates of small thiols with VS, Mal, and MS derivatives reported in literature. R is star‐PEG and R' is either star‐PEG thiol or PEG dithiol.

^b^ Gelation kinetics of 5 wt.% star PEG4‐X with star PEG4‐SH at pH 7 in 10 mM HEPES buffer at 25°C.

The thiol‐methylsulfone (MS) offers an alternate strategy for covalent and cytocompatible cross‐linking with intermediate reaction kinetics between the thiol‐Mal and the thiol‐VS reactions and leads to cross‐linked hydrogels within conditions used for cell culture [22]. The reaction between heteroaromatic ‐MS and ‐SH groups is a nucleophilic aromatic substitution (S_N_Ar), and forms aryl‐thioethers and methylsulfonate anion as the leaving group (Table 1). Under physiological conditions (HEPES buffer, pH 7–8), the MS group reacts with thiols to conversion > 90%, selectivity > 93% vs. amine groups, and gelation times of ≈1–3 min [22, 23]. The resulting thioether bond is stable against aqueous hydrolysis and enzymatic degradation (Table 1) [24]. In previous work, we explored the gelation of 4‐arm star PEG (PEG4) functionalized with three different heteroaromatic MS candidates, PEG4‐ArMS, where Ar varied between oxadiazole (OxMS), benzothiazole (BtMS), or tetrazole (TzMS) heteroaromatic rings. In 5 wt. % hydrogels, we demonstrated 3D cell encapsulation with > 92% viability after 7 days of culture (Table 1) [24]. The gelation kinetics were observed in the following order, fastest for OxMS, followed by TzMS and then for BtMS, which was attributed to the increasing electrophilicity of the heteroaromatic rin*g*, with TzMS derivative offering gelation in a few minutes and therefore, being the most interesting candidate for 3D cell encapsulation (Table 1) [24, 25].

**FIGURE 1:**
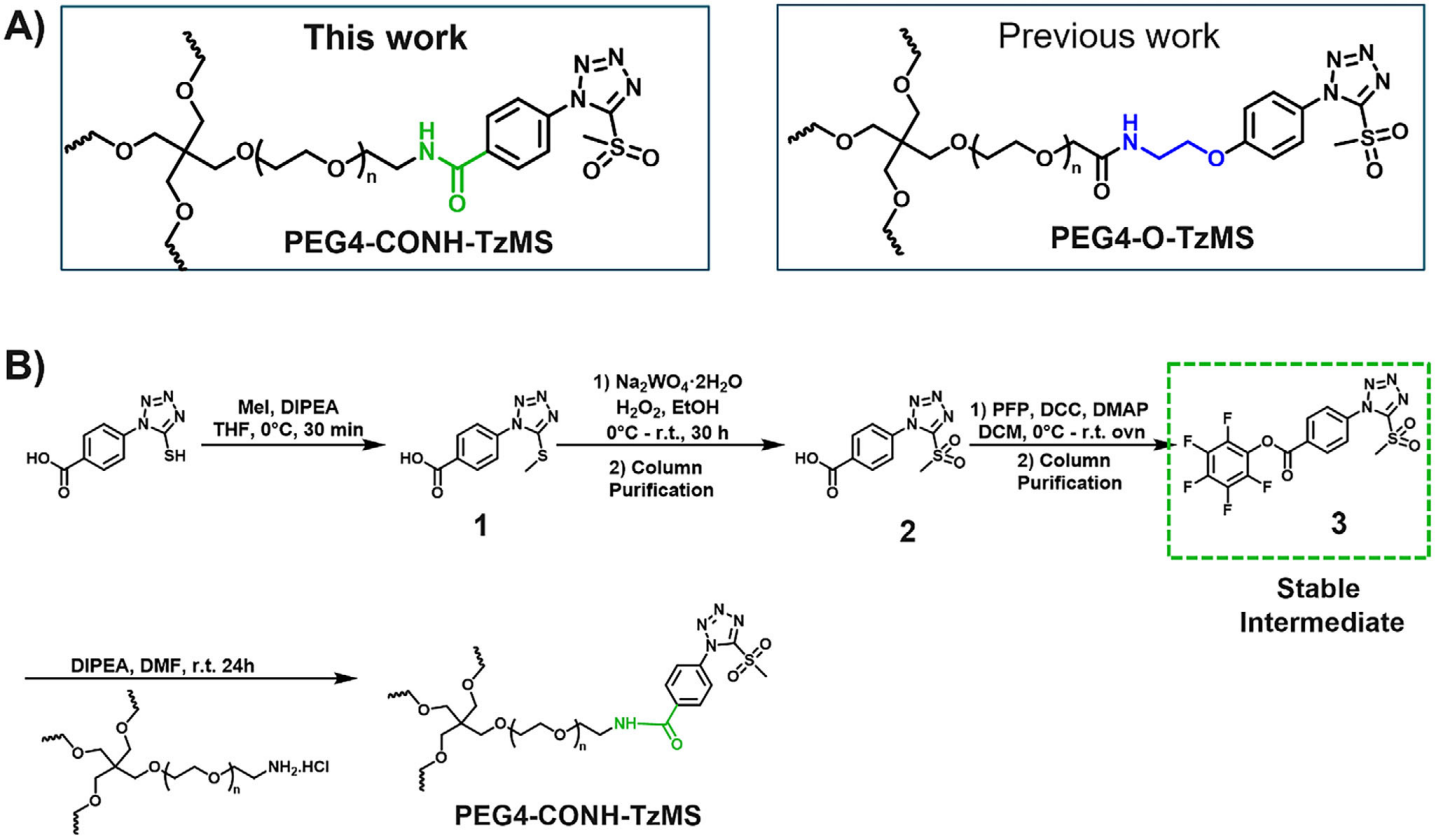
Synthesis of star‐PEG TzMS prepolymer. (A) Chemical structures of 4‐arm star‐PEG (PEG4) end‐functionalized with TzMS as synthesized in this work (PEG4‐CONH‐TzMS) and as previously reported PEG4‐O‐TzMS variant ref. (B) Synthesis pathway for PEG4‐CONH‐TzMS.

The synthesis protocol for PEG‐TzMS presents some challenges for scale‐up and wide use for cell encapsulation. One of the intermediates is difficult to purify and unstable during storage (Table 2) [26]. To address these shortcomings, we designed a pentafluorophenol (PFP)‐functionalized TzMS intermediate (compound 3, Figure 1B) for conjugation to amine‐containing polymers. This intermediate has desirable attributes of 1) attainment in one fewer reaction step, and 2) anchoring to polymeric chains through a stable amide bond. In addition, we did not expect to face stability issues. In our molecular design, we also considered the electrophilicity of the heteroaromatic ring, where the electron‐donating or electron‐withdrawing character of substituents at *p*‐position on the aryl ring is reported to influence the reaction kinetics of S_N_Ar reactions of the ArMS groups at physiological conditions [25]. Motiwala et al. quantified this effect in the reaction of TzMS derivatives with cysteine. A 2‐fold difference in the reaction rate was reported between the *p*‐OH (electrodonating) hydroxyl (*k* = 6.03 ± 0.55 M^−1^ s^−1^) vs. the *p*‐COOH (electrowithdrawing) derivative (*k* = 11.2 ± 0.46 M^−1^ s^−1^) (Table 1). The accelerated reaction kinetics were attributed to the inductive effect of the carboxylic acid group on the Tz ring, which reduces the electron density at the carbon atom next to MS [25]. In contrast, similar changes in the electronic character of the *p*‐substituent at BtMS derivatives led to orders‐of‐magnitude differences in the reaction rate [25].

**TABLE 2:** Comparison of cost and effort for synthesis of TzMS derivatives.

	Current Work	Previous Work
Structure of prepolymer	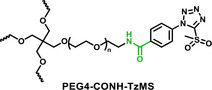	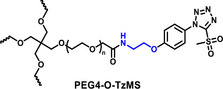
Key intermediate	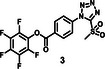	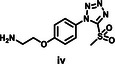
Reaction steps to attain the key intermediate	3	4
Column required for purifications	2	4
Stability of intermediate	Stable in storage in a dry state for > 6 months	14% decomposed after 15 min of purification from prep. HPLC.
Time to attain crude PEG4‐TzMS (days)	≈5	≈9
Materials costs (€/g intermediate) [1]	≈150	≈300
Stability of commercial PEG precursors	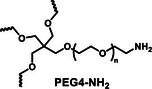 PEG4‐NH_2_ stable in storage	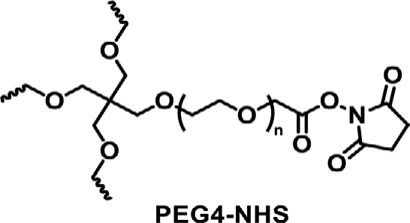 PEG4‐NHS is unstable in storage. Hydrolyzed over time

We hypothesized that the relatively lower sensitivity of TzMS reactivity to the electronic character of the *p*‐substituent and the expected higher overall reaction yield of the new synthesis route could facilitate access to TzMS functionalized pre‐polymers in larger scale while maintaining desirable crosslinking kinetics for cell encapsulation. In this work, we present the star poly(ethylene glycol)‐4‐(5‐(methylsulfonyl)‐1H‐tetrazol‐1‐yl)benzamide (PEG4‐CONH‐TzMS) hydrogel system bearing an electron‐withdrawing amide linker at the *p‐*position and study the influence of the *p*‐substituent on the properties of TzMS hydrogels by comparing it with the star poly(ethylene glycol)‐N‐(2‐(4‐(5‐(methylsulfonyl)‐1H‐tetrazol‐1‐yl)phenoxy)ethyl)acetamide (PEG4‐O‐TzMS) variant containing an electron‐donating linker (Figure 1A). We studied gelation kinetics, crosslinking degree, cytocompatibility and stability of the resulting hydrogels, and demonstrated encapsulation of single cells or spheroids in 3D cultures.

## 2 Results

### 2.1 Synthesis, Reactivity, and Stability of PEG4‐CONH‐TzMS

The synthesis route to PEG4‐CONH‐TzMS is shown in Figure 1B. Starting from the commercial precursor 4‐(5‐mercapto‐1H‐tetrazol‐1‐yl)benzoic acid, the synthesis involved first S‐methylation and then oxidation to methylsulfone derivative. The ‐COOH group in 4‐(5‐(methylsulfonyl)‐1H‐tetrazol‐1‐yl)benzoic acid (2) was activated to pentafluoro phenol (PFP) ester to facilitate the reaction with amine (‐NH_2_) terminated 4‐arm PEG. The intermediate 3 was obtained with 28% yield over three steps. This intermediate is stable in a dry state at 4°C for > 6 months (Figure 1B, Figure S1). The amine coupling reaction to PEG4‐NH_2_ proceeded with high yield (92.5%) at substitution degree > 95% at 400 mg scale (Figure 1B).

**FIGURE 2:**
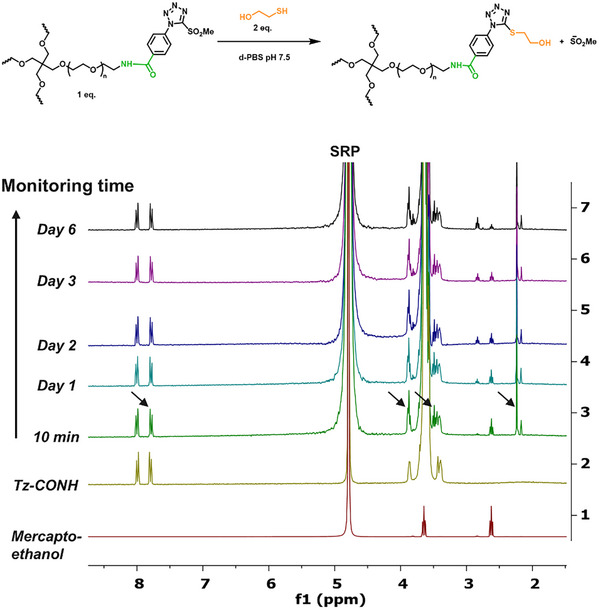
Reactivity and stability study of PEG4‐CONH‐TzMS with mercaptoethanol. ^1^H‐NMR of a solution of 1 mM PEG4‐CONH‐TzMS and 2 mM mercaptoethanol in deuterated‐PBS (pH 7.5). The spectrum at 10 min shows that the reaction was completed within this time. The spectra of the same mixture measured for up to 6 days confirm the stability of the resultant product.

The stability of PEG4‐CONH‐TzMS prepolymer in deuterated phosphate buffer saline (PBS) at pH 7.5 was investigated by ^1^H‐NMR spectroscopy (Figure S2). The PEG4‐CONH‐TzMS remained > 90% intact over 20 days of storage at 4°C. Next, we studied the reactivity of the PEG4‐CONH‐TzMS prepolymer with the model small molecule mercaptoethanol at a 2:1 thiol/MS molar ratio in deuterated‐PBS by ^1^H NMR. The reaction with PEG4‐CONH‐TzMS was indicated by the shifting of aromatic protons to slightly up field (7.89–7.79 ppm), appearance of two triplets in the aliphatic region at 3.89 ppm and at 3.50 ppm (formal overlapped with existing signals of CH_2_‐NH group of PEG and resulted in doubling the integral values) and appearance of a singlet at 2.23 ppm corresponds to the methyl sulfonate anion confirms the completion of TzMS/SH reaction (Figure 2). According to the spectrum, the reaction was completed within 10 min, i.e. before completion of the first NMR measurement. Last, we tested the stability of the reaction product during storage in deuterated‐PBS solvent at 4°C at different time points. No degradation was observed for 6 days (Figure 2). These studies confirm the aqueous stability of the reaction product at physiologically relevant conditions during the tested time scales.

### 2.2 Gelation Kinetics and Stability of PEG4‐CONH‐TzMS Hydrogels

**FIGURE 3:**
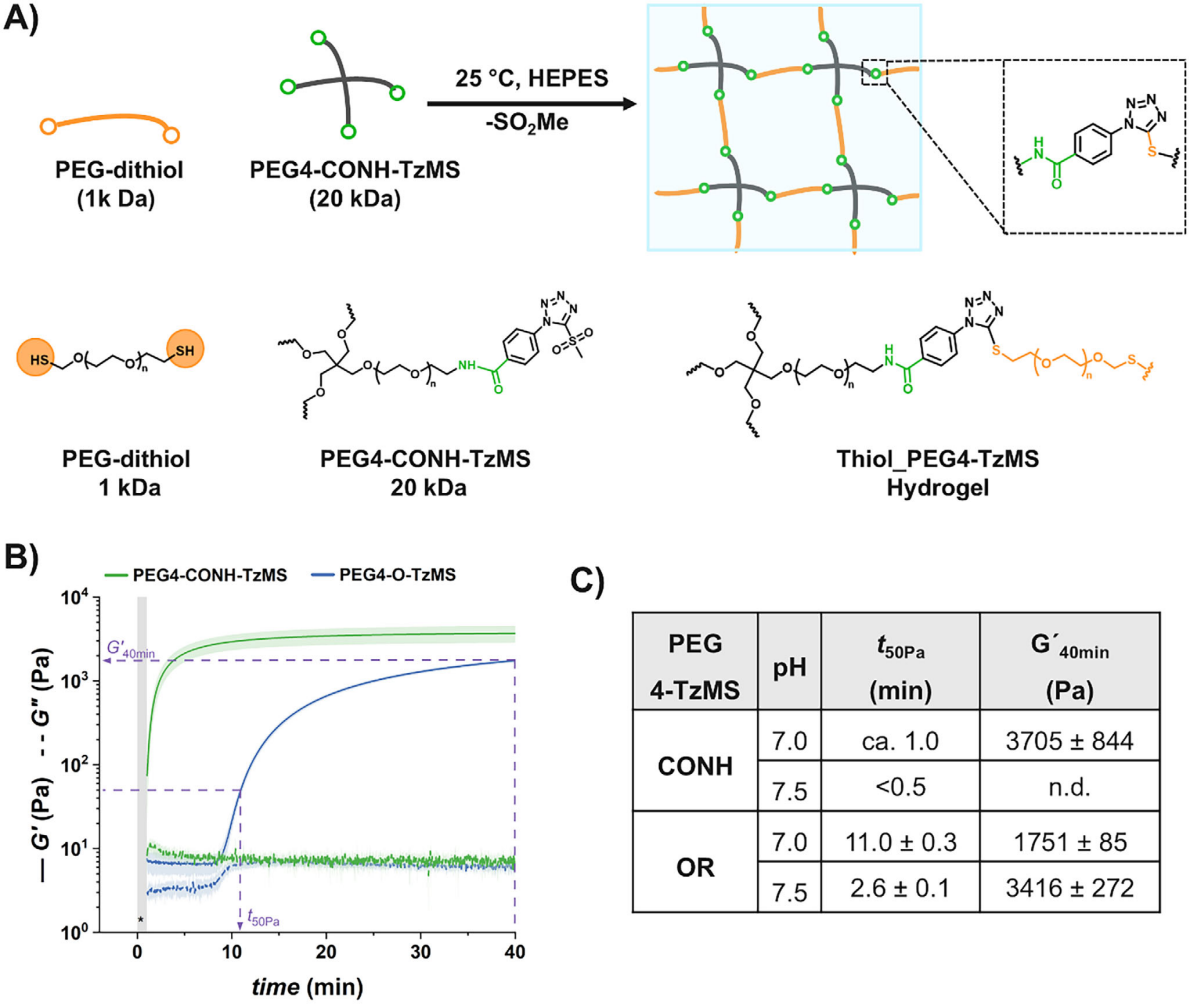
Crosslinking of PEG4‐X‐TzMS hydrogels and rheological characterization. (A) Schematic illustration of the crosslinking of PEG4‐CONH‐TzMS hydrogels with PEG‐dithiol crosslinker. (B) Representative curves of shear storage (G′) and loss (G″) moduli as a function of time (pH 7.0, T = 25°C). The grey region indicates the time for loading and mixing of the precursor solution until the start of the rheology measurement. (C) Values of t_50_Pa and G′_40 min_ G for the two hydrogels. Hydrogel composition: 1:1 MS/thiol precursors, 5 wt.% final polymer content, 50 mM HEPES buffer.

To form hydrogels with PEG4‐CONH‐TzMS, we used 1 kDa PEG‐dithiol as a crosslinker (Figure 3A). We monitored the gelation kinetics of the mixture in HEPES buffer (pH 7.0, 25°C) by in situ oscillatory shear rheology (Figure 3A). The evolution of the shear storage (G′) and loss (G″) moduli during 40 min after mixing the precursor solutions at 1:1 MS/thiol ratio is shown in Figure 3B. A steep increase in the storage modulus was observed during the first 4 min followed by a plateau value. This indicates fast and complete formation of the PEG4‐CONH‐TzMS hydrogel within a minute time scale (Figure 3C). To compare the values of the reaction time and the final mechanical properties of the hydrogels, we extracted the values of the time at which G′ reached 50 Pa (i.e., t_50Pa_) and the value of the G′ 40 min after mixing (i.e., G′ _40 min)_ (Figure 3B,C). The PEG4‐CONH‐TzMS hydrogels reached t_50Pa_ in ≈1 min and a final G′ of 3705 ± 844 Pa (Figure 3B,C). In comparison, the t_50_Pa and G′_40 min,_ measured for PEG4‐O‐TzMS were 11 min and 1751 ± 85 Pa, respectively.

**FIGURE 4:**
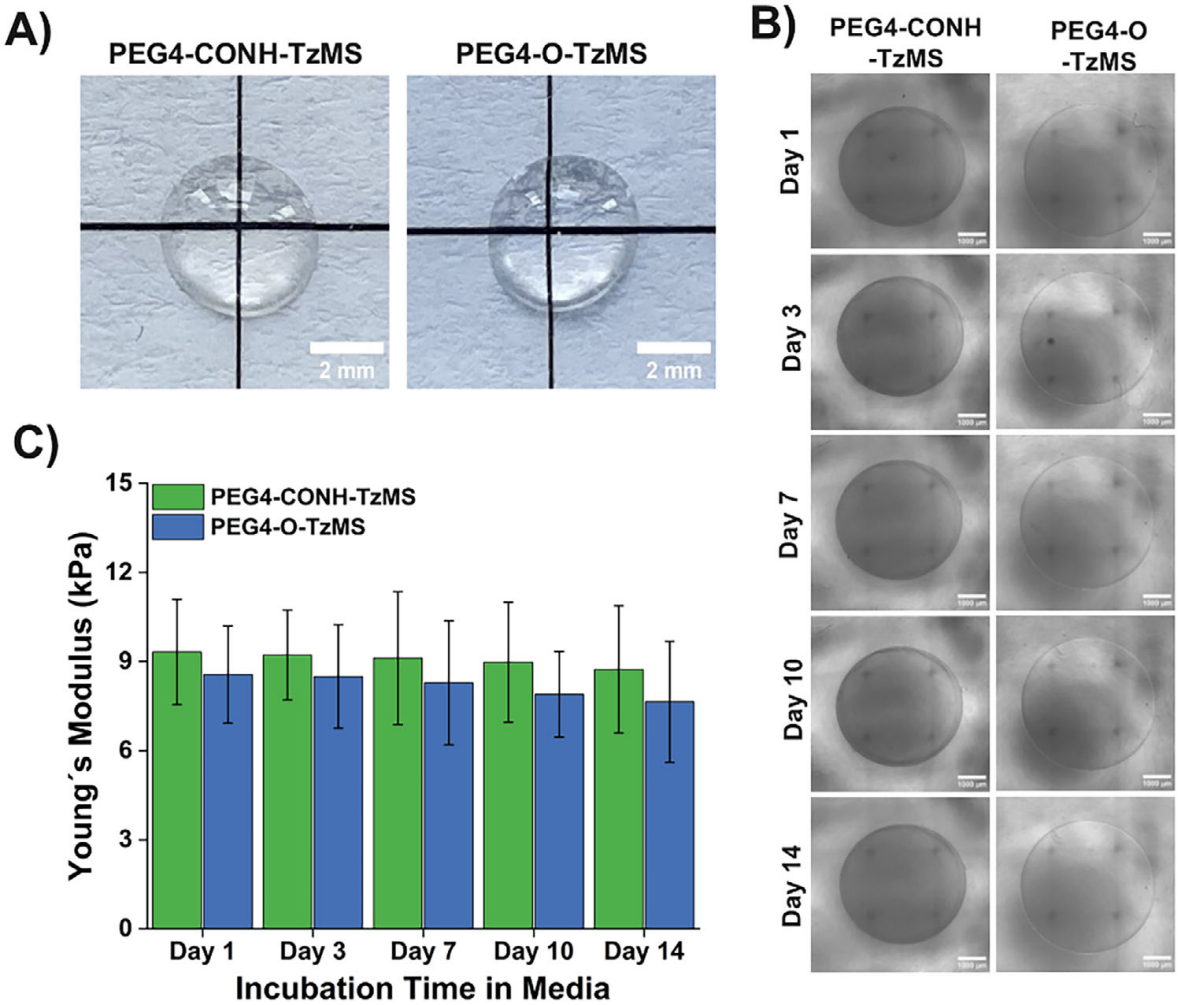
Quantification of stability of PEG‐TzMS hydrogels in cell culture medium. (A) Photograph of swollen PEG‐TzMS hydrogels in PBS showing their transparency. (B) Brightfield images of PEG‐TzMS hydrogels incubated in cell culture medium. (C) Young's modulus of incubated gels in cell culture medium. Bars represent mean ± standard deviation (SD) from three independent experiments, each with 4 technical replicates. Only fits with a coefficient of determination (R^2^) ≥ 0.95 were included in the analysis. Hydrogel composition: 1:1 MS/thiol precursors, 5 wt. % final polymer content, 50 mM HEPES buffer. Medium was RPMI with 10% FBS, pH 7.5, 37°C.

The PEG4‐CONH‐TzMS hydrogels (1:1 MS/thiol) obtained on gelation for 15 min (at pH 7.0, 37°C) were transparent and homogeneous, and qualitatively similar to previously reported PEG4‐O‐TzMS variant at macroscopic scale (Figure 4A) [27]. The stability of PEG4‐CONH‐TzMS (1:1 MS/thiol) hydrogels under standard culture conditions (i.e., RPMI media with 10% FBS, pH 7.5, 37°C) was investigated over time by brightfield imaging and nanoindentation. No significant changes in the dimensions of the hydrogels or the surface morphology were observed in brightfield images over > 15 days of incubation (Figure 4B,C).

These studies confirm the faster gelation kinetics (within a few minutes) and long‐term stability of PEG4‐CONH‐TzMS hydrogels vs. previously reported PEG4‐O‐TzMS counterparts under cell culture conditions.

### 2.3 Cell Encapsulation in PEG4‐CONH‐TzMS Hydrogels

**FIGURE 5:**
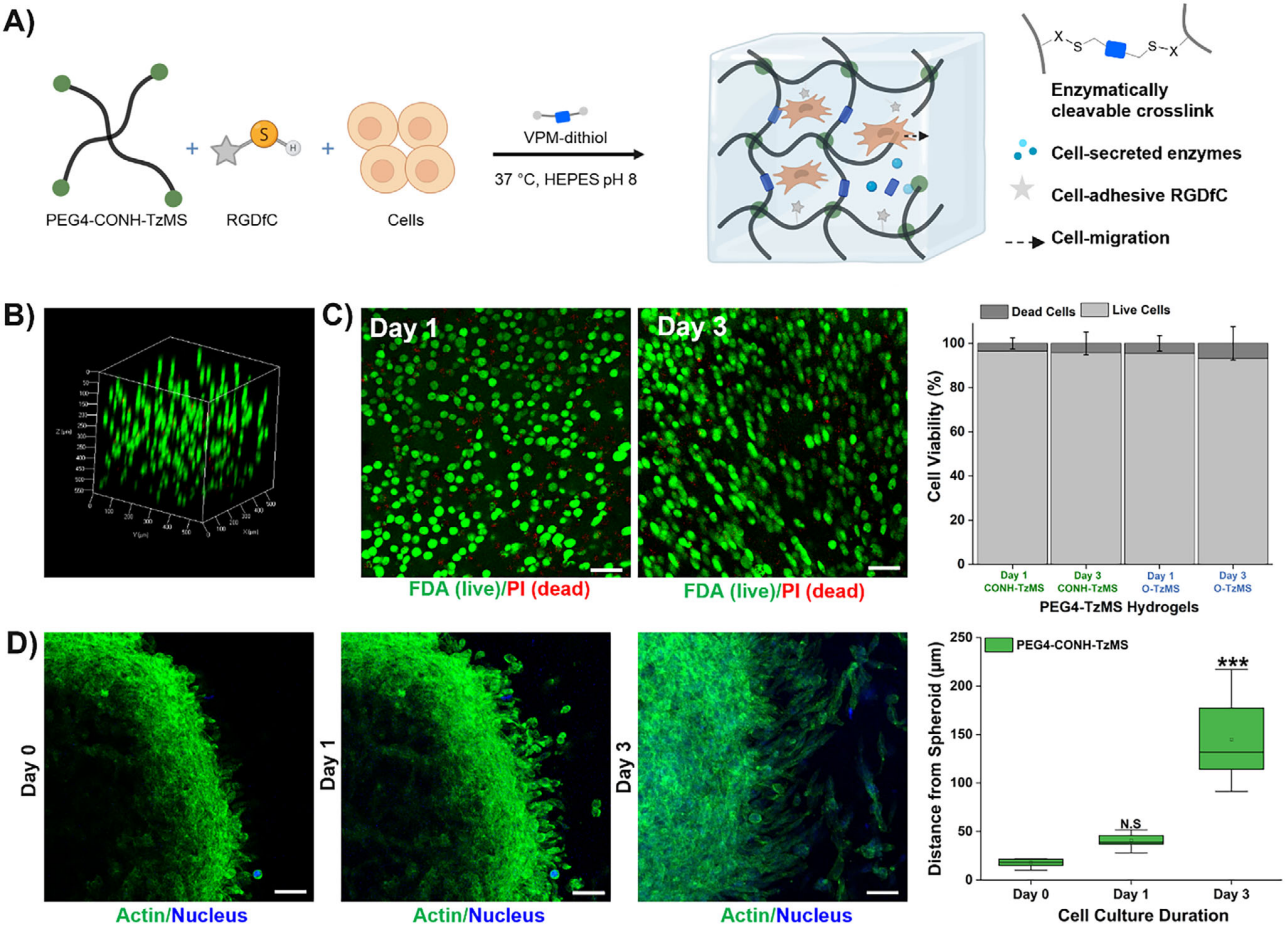
Cell encapsulation in PEG‐TzMS hydrogels. (A) Schematic representation of encapsulation of enzymatically cleavable PEG hydrogels modified with cell adhesive peptide (RGD). (B) Z‐stack fluorescence image of L929 fibroblasts encapsulated for 1 day in enzymatically cleavable PEG hydrogels. Hydrogel composition: 4 wt. % PEG4‐CONH‐TzMS, 1 mM RGD cell adhesive peptide, 3.5 mM VPM‐dithiol peptide, 10 mM HEPES buffer pH 8.0. The live cells were stained with fluorescein diacetate (FDA, green). (C) Fluorescence images of a live/dead assay of fibroblasts encapsulated for 1–3 days in the PEG4‐CONH‐TzMS hydrogels (The scale bar in C is 50 µm). The live cells were stained with fluorescein diacetate (FDA, green) and dead cells were stained with propidium iodide (PI, red) (left). The viability was quantified from fluorescence z‐stack images from an FDA/PI based assay of cells after 1 or 3 days of encapsulation in PEG hydrogels (right). (D) Fluorescence images of L929 cells migration from spheroids encapsulated in PEG4‐CONH‐TzMS hydrogels as a function of time (The scale bar in D is 50 µm). The fluorescence images were constructed from confocal z‐stack images of scaffolds taken after staining of encapsulated cells with live F‐actin (Biotracker 497, green) and nucleus (hoechst, blue) markers (left). Quantification of migration distance over time (right). The data is expressed as means ± SD and statistically analyzed by using one‐way ANOVA with Tukey‐Kramer post hoc test ^***^ *p* < 0.0001 comparing spreading areas on day 1 and 3, to day 0.

The suitability of PEG4‐CONH‐TzMS for cell culture was tested by encapsulating L292 fibroblasts as model cells in the 3D hydrogels and evaluating the viability and migration of single cells and spheroids, respectively. The PEG4‐CONH‐TzMS prepolymer was biofunctionalized with the cell adhesive cyclo‐RGDfC peptide in a preincubation step, and then mixed with an L929 fibroblast suspension, followed by crosslinking with a matrix metalloproteinase (MMP) cleavable peptide containing two cysteine groups for reaction with the Tz group (VPM) (Figure 5A). A composition of 4 wt.% TzMS, 1 mM RGD peptide, and 3.5 mM VPM was used. The concentrations were chosen to offer a good compromise between integrin binding sites and crosslinking degree via reaction of the remaining ‐MS groups with VPM [28]. Upon mixing, solution viscosity remained low for > 30 sec, enabling mixing by pipetting at low shear forces. The mixture was left for 15 min before adding medium and imaging at different time points (1–3 days). A uniform distribution of cells throughout the thickness of the hydrogel was observed in the confocal Z‐stack images (Figure 5B). The live/dead assays performed on cells encapsulated for 1 day in PEG gels showed > 90% viability when crosslinked with PEG4‐CONH‐TzMS proving the cytocompatibility of our material (Figure 5C). The viability remained unaffected in longer cell cultures. This high value of cell viability is consistent with previous studies with PEG4‐O‐TzMS variant (Figure 5C) [24, 27]. To evaluate the growth and functionality of cells encapsulated in PEG hydrogels, migration assays were performed [22, 29, 30]. Encapsulated fibroblast spheroids showed cell spreading and active migration (≈50 µm/day) over 3 days of culture, indicating both recognition of cell‐adhesive RGD ligand and cleavage of the enzymatically degradable peptide bonds provided by the VPM crosslinker (Figure 5D). These studies corroborate the cytocompatibility of PEG4‐CONH‐TzMS hydrogels for 3D cultures.

## 3 Discussion

Hydrogels for cell encapsulation require efficient and selective crosslinking mechanisms with crosslinking kinetics that match the duration of the encapsulation step at reaction conditions (buffer, pH) appropriate for living cells. Ideally, precursor materials can be synthesized in a small number of steps and are stable during storage. In this work, we show that PEG4‐CONH‐TzMS can be synthesized in 4 steps with an overall yield of 26% at a 400 mg batch size. The process required 2 column purification steps (Figure 1). This represents a significant improvement in the synthetic protocol vs. our previously reported PEG4‐O‐TzMS, which required 5 steps and 4 column purifications for an overall yield of 32% at 600 mg batch size (Figure S3 and Table 2) [24]. The intermediate compounds in the synthesis of PEG4‐CONH‐TzMS are stable and can be stored long time, whereas the amine intermediate in the synthesis of PEG4‐O‐TzMS was highly unstable and had to be consumed immediately [26]. The stable PFP‐Tz intermediate 3 can be used to derivatize ─NH_2_ and ─OH groups in polymeric backbones. For demonstration, we tested this reaction on 40 kDa PEG8‐NH_2_, 31 kDa poly(vinyl alcohol), and Gelatin type A 300 bloom. To further showcase the utility of the new precursor, intermediate 3 was also derivatized with carboxylic acid‐containing backbone (15 kDa Heparin) by introducing a small amine‐terminated linker into intermediate 3. In all cases, reaction yields between 65 and 85% were obtained following straightforward synthetic routes (Figure S4 and see supporting information for details) [31, 32, 33]. These experiments substantiate that the changes introduced in the synthetic pathway to overcome the lower stability of previously reported reaction intermediates are applicable for the synthesis of various polymers.

Reported data show that the electrophilicity of the heteroaromatic ring in ArMS increases the reactivity with thiol groups and decreases the stability of the ArMS molecule and the reaction product [24, 25]. ^1^H‐NMR analysis for the stability of PEG4‐CONH‐TzMS prepolymer in deuterated PBS at pH 7.5 showed a 10% decomposition in 20 days (Figure S2). This result is similar to the reported stability of PEG4‐O‐TzMS, indicating that the changes in the *p*‐substituent did not significantly affect the stability of the hydrogel precursor. In terms of reaction conversion, a quantitative MS‐thiol reaction was observed after 10 min of reaction of PEG4‐CONH‐TzMS with a low molecular weight thiolated molecule in a deuterated PBS buffer of pH 7.5. The reaction product was stable in PBS buffer solution for over 6 days (Figure 2). Together with the stable mechanical properties observed for the hydrogel in nanoindentation experiments, our results confirm that the EWG substituent on the TzMS does not interfere with the stability of the thioether product.

Under the same gelation conditions, PEG4‐CONH‐TzMS showed an 11‐fold faster gelation kinetics and reached a 2‐fold higher storage modulus (after 40 min) than PEG4‐O‐TzMS variant (Figure 3B,C, and Table 1). These results confirm that the electron‐withdrawing character of the amide group facilitates the nucleophilic aromatic substitution (S_N_Ar) reaction, presumably because it decreases the electron density at the MS‐carbon. The gelation time observed for PEG4‐CONH‐TzMS hydrogels (60 – 90 s) at 50 mM HEPES buffer at pH 7, allows homogenous mixing of cells during the cell encapsulation process, while maintaining a crosslinking degree and mechanical properties that match those of the faster PEG‐Mal or PEG‐OxMS systems. PEG4‐CONH‐TzMS yields hydrogels with high hydrolytic and enzymatic stability, supporting the growth and migration of encapsulated fibroblasts.

## 4 Conclusions

The selection of the best possible aryl‐MS derivative for a hydrogel for cell encapsulation experiment depends upon the duration and experimental conditions of the encapsulation steps (buffer, pH), which need to be matched by the kinetics of the gelation, and the required final properties of the hydrogel (stiffness, stability). Our previous work with 5 wt.% PEG4‐X‐MS (X = Ox, Btz, Tz) demonstrated that TzMS is an adequate ArMS derivative for cell encapsulation, which provides gelation kinetics in the range of minutes (7 min) in HEPES buffer at pH 7. By varying the character or the substituent at the *p*‐position of the aryl ring (EWG vs. EDG), this study shows that the reactivity of the TzMS derivative vs. thiol groups can be tuned, and this translates into hydrogels with gelation kinetics in a useful range (i.e., ≈60 sec) in HEPES buffer at pH 7.0. The 11‐fold increase in gelation kinetics and 2‐fold increase in stiffness are important for cell encapsulation because it allows homogenous cell distribution in mechanically stiff and enzymatically stable hydrogels. Moreover, we have streamlined the synthesis of PEG‐TzMS, with enhanced shelf life of key intermediate (3) (months vs. minutes), which not only enhanced the potential for cost‐effective and large‐scale production of TzMS‐terminated star‐PEGs but also offered straightforward extension to other biocompatible polymeric backbones.

## 5 Experimental Section

### 5.1 Preparation of TzMS PEG Hydrogels

The synthesis of PEG4‐CONH‐TzMS precursor was described in Figure 1B and supporting information. The synthesis of PEG4‐O‐TzMS has been reported in the literature (Figure S3) [22]. Precursor solutions of 20 kDa PEG4‐TzMS (‐CONH/OR) (100 mg mL^−1^, 10 wt.%) were prepared by dissolving the lyophilized polymer in HEPES buffer (50 mM). The pH of the solution was adjusted to 7.0 or 7.5 using 1.0 m NaOH and measured by Eutech Elite pH Spear (Thermo Scientific). Solution of PEG‐dithiol (1 kDa, 0.88 wt.% or 0.94 wt.%) was also prepared in HEPES buffer (50 mM). The hydrogel precursor solutions were mixed in equal volumes (1:1), to obtain hydrogels with 5 wt.% of final polymer content following reported protocols [24, 27]. The composition of precursor solutions was kept constant for all hydrogel characterization and stability experiments, unless otherwise specified.

### 5.2 Oscillatory Shear Rheology of TzMS PEG Hydrogels

The rheological characterization of the hydrogel crosslinking was performed on a Discovery HR‐3 rheometer (TA Instruments, USA) equipped with a 12 mm parallel plate geometry and a Peltier stage temperature control system. Hydrogel precursor solutions were prepared immediately before the measurement as described above. 18 µL of a PEG4‐TzMS (‐O or ‐CONH, 10 wt.%) solution was loaded on the lower plate of the rheometer. To this, 18 µL of PEG‐2SH (0.88 or 0.94 wt.%) was added, and the solutions were carefully mixed with a pipette tip. The upper plate was lowered to a final gap of 300 µm, the sample was sealed with silicon oil and covered with a metal cover to avoid evaporation and disturbances during the measurement. The time sweep measurement started 60 s after the sample loading. Oscillatory shear rheology was performed over 40 min using 1.0% strain and 1 Hz frequency at T = 25°C. The gelation time was compared using the timepoint when the storage modulus G′ has reached a value of 50 Pa (t_50_ Pa), while the storage modulus after 40 min of crosslinking was used to compare the mechanical properties of the hydrogels (G′ 40 min) [27]. Note that 60 s before the rheometer launch were taken into account to calculate the final stiffness of the samples.

### 5.3 Brightfield Imaging of PEG4‐TzMS Hydrogels

The qualitative structural integrity of the hydrogels was followed by taking brightfield images using (Zeiss CD7) microscope. The PEG4‐TzMS (─CONH/─O) hydrogels were prepared in a 24‐well plate format by dispensing 10 µL of precursor solution per well. Brightfield images of hydrogels incubated at 37°C in RPMI 1640 medium supplemented with 10% FBS and 1% penicillin/streptomycin were taken at regular time intervals.

### 5.4 Nanoindentation Measurements

The qualitative changes in the mechanical properties of the hydrogels were characterized by nanoindentation using the Pavone Nanoindenter (Optics11, Amsterdam, Netherlands), equipped with a spherical probe (48 µm radius) mounted on a cantilever with a spring constant of 0.50 N m^−1^. Hydrogels were prepared in a 384‐well plate format by dispensing 15 µL of precursor solution per well. Indentation measurements of hydrogels incubated at 37°C in RPMI 1640 medium supplemented with 10% FBS and 1% penicillin/streptomycin were measured using indentation‐control mode at a constant speed of 1 µm s^−1^. Data was analyzed with Data Viewer software (version 2), and Young's modulus was calculated using the Hertzian contact model.

### 5.5 3D Cell Encapsulation

The L929 fibroblasts (ATCC) were cultured at 37°C and 5% CO_2_ in RPMI 1640 medium (Gibco, 61870−010) supplemented with 10% FBS (Gibco, 10270) and 1% P/S (Invitrogen) as established in the literature [28, 34]. The L929 cells were used between passages 4 and 10.

The cells were encapsulated in 3D PEG hydrogels by adapting previously reported protocols [20]. Precursor solutions of 20 kDa PEG4‐TzMS (‐CONH/‐O) (100 mg mL^−1^, 10 wt.%) were prepared by dissolving the lyophilized polymer in sterile HEPES buffer (10 mM, pH 8.0) inside a sterile laminar flow and used directly without further filtration. Solutions of cyclo(RGDfC) (2.9 mg mL^−1^, 5 mM) and VPM peptide (GCRDVPMSMRGGDRCG, 29.7 mg mL^−1^, 17.5 mM) were also prepared in sterile HEPES buffer (pH 8.0). These concentrations were kept constant during all cell experiments. The L929 cells (1 × 10^6^ mL^−1^ cells) in complete RPMI media were directly suspended in the PEG precursor solution for polymerization. The PEG4‐TzMS stock solution (4 µL, 4 wt.%) was mixed with cyclo(RGDfC) (2 µL, 1 mM) and incubated for 30 min at 37°C. The fibroblast cell suspension (2 µL, 2 × 10^5^ cells mL^−1^) was added to the above solution. The resulting mixture (8 µL) was placed in an Ibidi 15‐well angiogenesis µ‐Slide, and VPM peptide (2 µL, 3.5 mM) was added and carefully mixed with a pipette tip. The mixture was allowed to polymerize for 15 min, followed by addition of complete RPMI medium (50 µL), yielding hydrogels with a thickness of 800 µm. The cells were maintained for 1−3 days at 37°C and 5% CO_2_, and media was substituted by fresh medium every 24 h during cell culture.

### 5.6 Live/Dead Assay

L929 fibroblasts were cultured in PEG hydrogels for 1 or 3 days, and the cell culture medium was removed. Samples were incubated for 5 min with fluorescein diacetate (40 µg mL^−1^) and propidium iodide (30 µg mL^−1^) in PBS and washed twice with PBS. The 3D hydrogels were imaged with an LSM 880 confocal microscope (Zeiss). For each sample, at least five independent z‐stacks at 20x magnification were analyzed (≈1000 cells per sample). Live and dead cells were counted manually in each slice of the z‐stack to calculate the percentage viability of each sample by using Zen Blue (Version 3.5, Zeiss). Cell viability data were expressed as means ± SD.

### 5.7 Cell Migration Assay

Spheroid encapsulated PEG hydrogels were prepared by adapting previously reported protocols [22, 29, 30]. For spheroid culture, a fibrin clot of the fibroblast L929 cell line was prepared by following the literature reports. Briefly, one pellet of 1 × 10^7^ mL^−1^ cells was dissociated in fibrinogen (10 mg mL^−1^ in PBS), and 2 µL drops (2.7 × 10^4^ cells/spheroid) were placed on a hydrophobic Sigmacote‐coated glass slide. Next, 1 µL of thrombin solution (5 UN mL^−1^ in PBS) was mixed with each drop of fibrinogen, and the cells were placed in an incubator for 15 min to get a fibrin clot. Finally, the complete RPMI medium (2 µL) was mixed with cyclo(RGDfC) modified PEG4‐CONH‐TzMS solution (6 µL, as described above) and added (8 µL) to the µ‐Slide. One fibrin clot was added to each well, followed by the addition of VPM peptide (2 µL, 3.5 mM), and allowed to gelate for 15 min at 37°C. The cells were maintained for 1−3 days at 37°C and 5% CO_2_, and the media were substituted with fresh medium every 24 h during cell culture. The encapsulated fibroblasts labeled with live cell markers, i.e., Biotracker 497 Green Actin Live (1:500, Sigma) and Hoechst (1:500, Thermofisher) were imaged directly. The spheroid‐encapsulated hydrogels with unlabeled fibroblasts were fixed with 4% PFA solution for 2 h at room temperature and washed with PBS. Samples were incubated overnight with 0.01% Triton X‐100 in DPBS containing ActinGreen (1:50, ThermoFisher) to stain actin fibers and with NucBlue (1:50, ThermoFisher) for staining the nucleus. Samples were washed twice with PBS, and z‐stacks of stained samples were acquired using LSM 880 confocal microscope (Zeiss). Migration distance of cells from the spheroid was defined as the distance of the apical tip of a leading cell to the center of the spheroid. This distance was measured manually in all directions in a z‐stack (≈150−200 cells/samples) by using the Zen‐blue software to calculate the average distance covered by cells. Cell migration data are expressed as means ± SD and statistically analyzed by using a one‐way ANOVA with a post hoc Tukey's test.

### 5.8 Statistical Analysis

All experiments were composed of at least three independent experimental repeats. Data is expressed as mean ± SD. Statistical analysis for parametric results in cell migration assay data is established by using one‐way ANOVA with a post hoc Tukey test.

## Conflicts of Interest

The authors declare no conflict of interest.

## Supporting information




**Supporting File**: mabi70161‐sup‐0001‐SuppMat.docx.

## Data Availability

The data that support the findings of this study are available in the supplementary material of this article.
