# Supplementary material for: Thiol‐Methylsulfone Crosslinked Hydrogels for Cell Encapsulation: Molecular Scale Modulation of Physiochemical Properties
Source: Macromol Biosci. 2026 Feb 24;26(2):e00627. doi: 10.1002/mabi.202500627 (PMC12931027; doi:10.1002/mabi.202500627)
Supplement: Supplementary file 1 — Supporting File: mabi70161‐sup‐0001‐SuppMat.docx. [file MABI-26-e00627-s001.docx]

Supporting Information

**THIOL-METHYLSULFONE CROSSLINKED HYDROGELS FOR CELL ENCAPSULATION: MOLECULAR SCALE MODULATION OF PHYSIOCHEMICAL PROPERTIES**

Hafiz Syed Usama Bin Farrukh,^(1,2)^ Aleeza Farrukh,^(1)^ Syuzanna Hambardzumyan,^(1)^ Therese Steudter,^(1,2)^ Samuel Pearson,^(1)^ Aránzazu del Campo*^(1,2)^

(1) INM – Leibniz Institute for New Materials, Campus D2 2, 66123 Saarbrücken, Germany

(2) Saarland University, Chemistry Department, 66123 Saarbrücken, Germany

**(*) Corresponding author:**

Prof. Dr. Aránzazu del Campo: Tel: +49(0)681-9300-510, Fax +49(0)681-9300-223,

e-mail: [aranzazu.delcampo@leibniz-inm.de](mailto:aranzazu.delcampo@leibniz-inm.de) ORCID: 0000-0001-5725-2135

# **1. Chemical synthesis**

## **1.1. Materials and methods**

4-(5-mercapto-1H-tetrazol-1-yl)benzoic acid (> 95%), pentafluorophenol (> 98%) were purchased from TCI (Belgium). Iodomethane (99.5% contains copper as stabilizer), sodium tungstate dihydrate (≥ 99%), hydrogen peroxide (30 wt.% in water), 2-Mercaptoethanol (≥ 99%), diisopropyl ethyl amine (≥ 99%), 4-(Dimethylamino) pyridine (≥ 99%), and N,N′-Dicyclohexylcarbodiimide (≥ 99%) were purchased from Sigma Aldrich (Germany). 4-arm PEG Amine, HCl salt MW 20000 (≥ 99%) and 4-arm PEG succinimidyl carboxymethyl ester MW 20000 (≥ 96.7%) were purchased from JenKem technology (USA). All the solvents were either ACS reagent grade or HPLC grade purchased from VWR or J.T. Baker (Germany). All the other chemicals were reagent grade purchased from TCI (Belgium) or Sigma Aldrich (Germany) and were used without further purification. Thin layer chromatography (TLC) plates (ALUGRAM® SIL G/UV_254_) were purchased from Macherey-Nagel, (Germany). Column chromatography was carried out on silica gel 60 (70–230 mesh) from Merck (Germany). Synthesized polymer was purified by dialysis using Spectra/Por 3 dialysis tubing (molecular weight cut-off MWCO= 3.5 kDa) from Spectrum Inc against water and acetone.

The ^1^H-NMR and ^13^C-NMR spectra were recorded with a Bruker Avance 300 MHz from Bruker (Massachusetts, USA) at 298 K and the solvent residual peak (S.R.P.) was employed as internal reference. Deuterated solvents were purchased from Deutero GmbH (Germany).

Analysis and purification of compounds were performed with HPLC JASCO 4000 (Japan) equipped with a diode array, UV-vis detector and fraction collector. Semi-preparative (250 × 25 mm) runs with a flow of 10 mL min^-1^ were conducted using Reprosil C18 columns while analytical (250 × 5 mm) runs with a flow of 1 mL min^-1^ were conducted using Reprosil C18 columns. Solvent gradients were used a combination of the following eluents, with typical run of 45 minutes, with solvent A (MilliQ water + 0.1% TFA) and solvent B (95% ACN / 5% MilliQ water + 0.1% TFA). Mass of synthesized compounds were recorded on 6545 Accurate Mass Quadrupole Time-of-Flight (LC/QTOF- MS) with electrospray ionization from Agilent (California, USA).

# **1.2. Chemical synthesis protocols**

*1.2.1. Synthesis of 4-(5-(methylthio)-1H-tetrazol-1-yl)benzoic acid (1):*

Compound 1 was synthesized by modifying previously reported protocol.^1^ 4-(5-mercapto-1H-tetrazol-1-yl)benzoic acid (3.0 g, 13.5 mmol, 1 equiv.) was dissolved in anhydrous THF (30 mL) under nitrogen atmosphere and the solution was cooled to 0 °C. N, N-Diisopropylethylamine (DIPEA, 5.87 mL, 33.75 mmol, 2.5 equiv.) was added to the above solution and the reaction mixture was allowed to stir for 5 minutes. To this turbid solution iodomethane (1.68 mL, 27.0 mmol, 2.0 equiv.) was added dropwise over a period of 10 min and the reaction mixture was allowed to stir at 0 °C for 30 minutes. The progress of reaction was monitored by TLC (DCM/MeOH/AcOH 9:1:0.02 v/v) every 15 minutes. After complete consumption of starting reactant (~ 30 min) the solvent was removed under vacuum, the residue was suspended in water and acidified with 1N HCl, the suspension was sonicated for 5 minutes and filtered under vacuum. The resulted off-white solid was washed three times with water and 1 time with n-hexane and air dried to afford the crude product in high yield (2.99 g, 94 %) which was used in the next step without further purification. The reported values in the literature are aligned with the spectroscopic characterization data.

ESI-MS+ (C_9_H_8_N_4_O_2_S): 237.0437 [M + H].

^1^H-NMR (300 MHz, DMSO-*d*_6_) δ (ppm) = 13.45 (s, 1H, -COOH), 8.19 (d, *J* = 8.5 Hz, 2H, -CHAr), 7.82 (d, *J* = 8.4 Hz, 2H, -CHAr), 2.80 (s, 3H, -SMe).

^13^C NMR (75 MHz, DMSO-*d*_6_) δ (ppm) = 166.15, 155.29, 136.39, 132.41, 131.00 (2C), 124.29 (2C), 15.32.


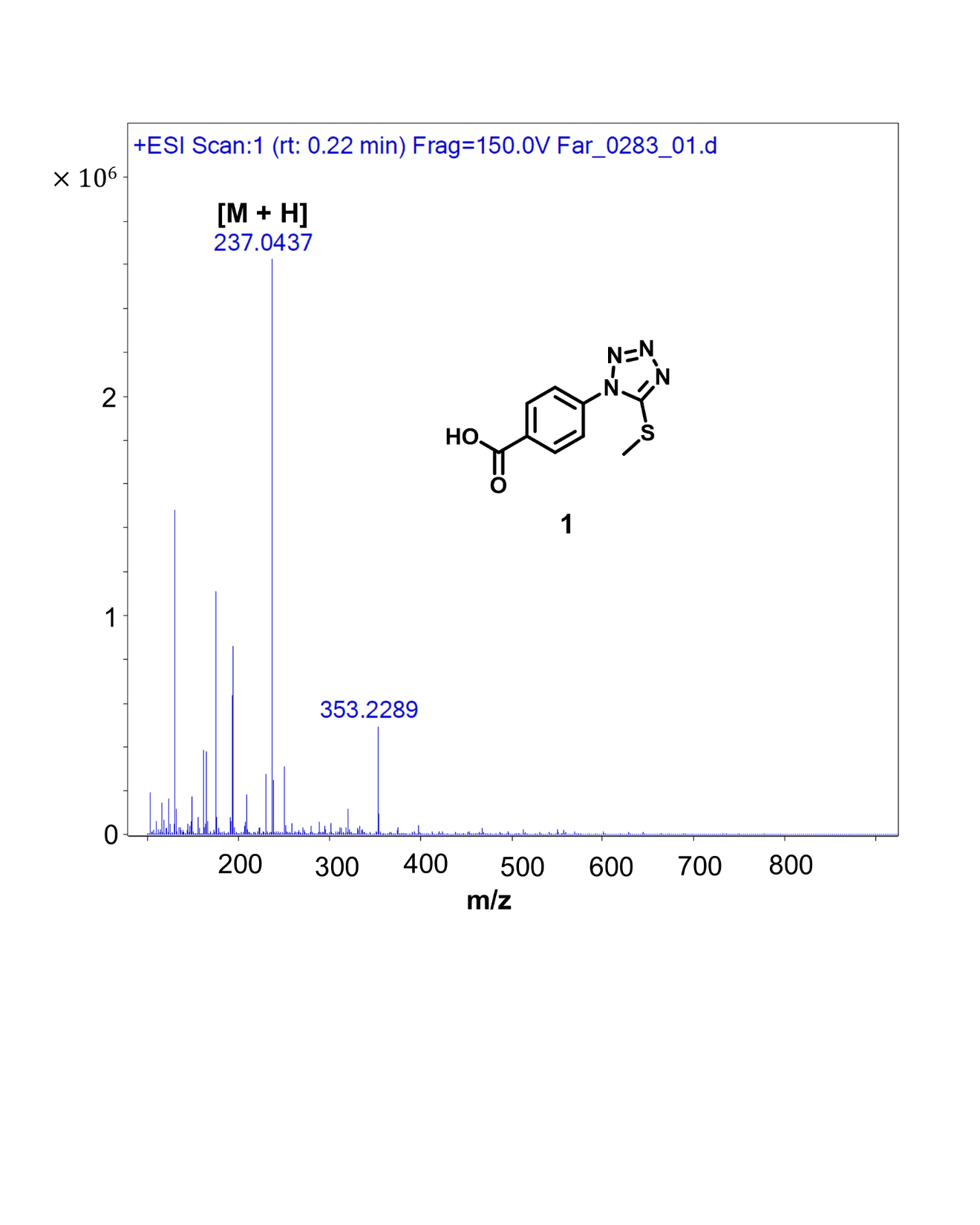


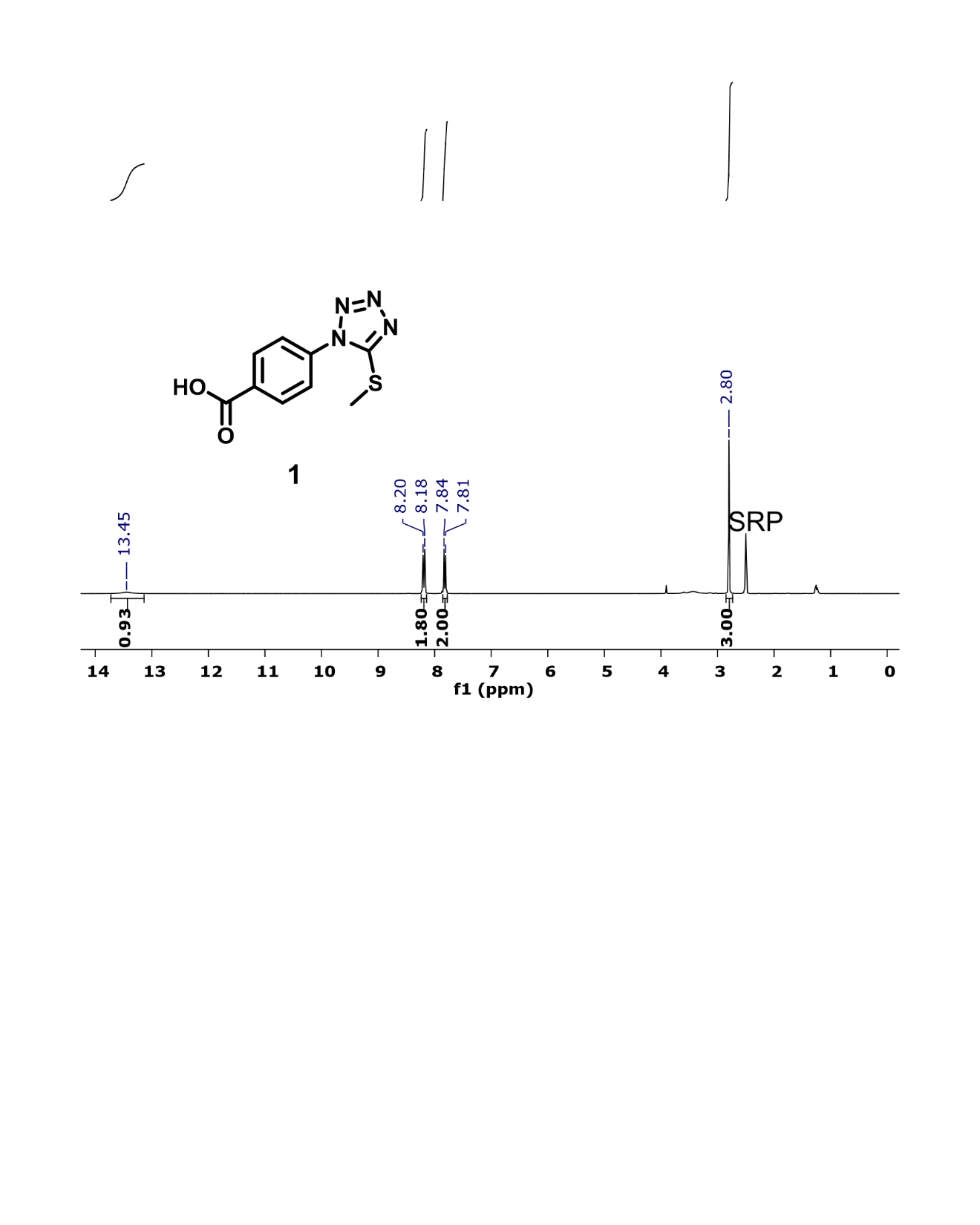


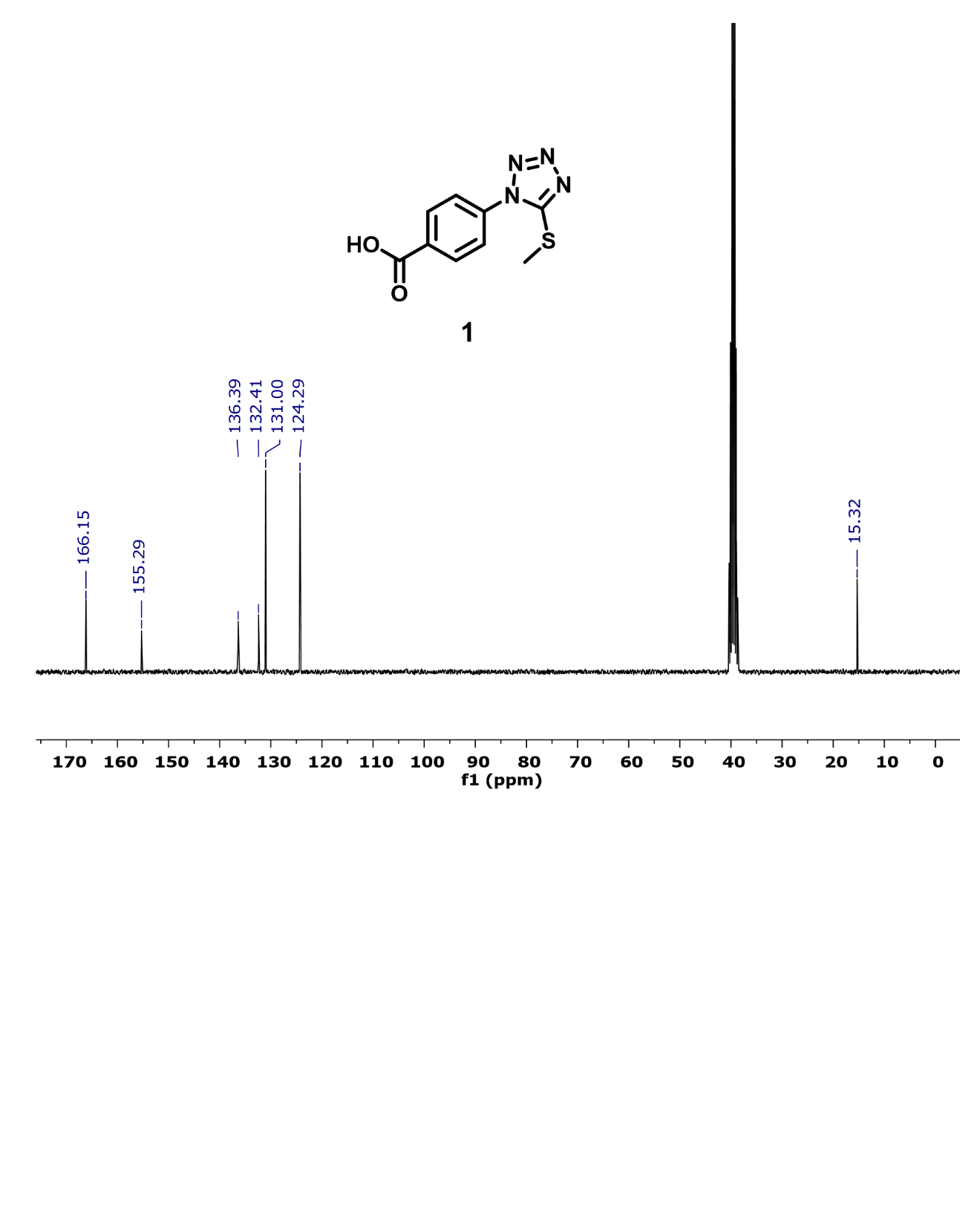


*1.2.2. Synthesis of compound 4-(5-(methylsulfonyl)-1H-tetrazol-1-yl)benzoic acid (2):*

Compound 2 was synthesized by modifying previously reported protocol.^1^ 4-(5-(methylthio)-1H-tetrazol-1-yl)benzoic acid (a, 0.4 g, 1.69 mmol, 1 equiv.) was suspended in 15 mL of absolute ethanol and the resulting mixture was cooled to 0 °C, at which point sodium tungstate dihydrate (0.28 g, 0.85 mmol, 0.5 equiv.) was added. To this colorless suspension hydrogen peroxide (30% w/w in H_2_O, 7 mL) was added dropwise over a period of 1 h. After complete addition the mixture was sonicated for 5 min and allowed to stir at room temperature for 30 h. The progress of reaction was monitored every 15 h by TLC (DCM/MeOH/AcOH 9:1:0.02 v/v/v). When all the stating reactant was consumed the ethanol was removed under vacuum, and the remaining aqueous solution was extracted with DCM and purified by column chromatography by using DCM and MeOH (95:5) as eluent to give pure product (0.195 g, 43 %) as a white solid. The reported values in the literature are aligned with the spectroscopic characterization data.

ESI-MS+ (C_9_H_7_N_4_O_4_S): 267.0199 [M - H]^-^

^1^H NMR (300 MHz, DMSO-*d*_6_) δ (ppm) = 8.17 (d, *J* = 8.5 Hz, 2H, -CHAr;), 7.82 (d, *J* = 8.6 Hz, 2H, -CHAr;), 3.69 (s, 3H, -SO_2_Me).

^13^C NMR (75 MHz, DMSO-*d*_6_) δ (ppm) = 167.22, 154.20, 136.51, 135.22, 130.17(2C), 126.16(2C), 43.96.


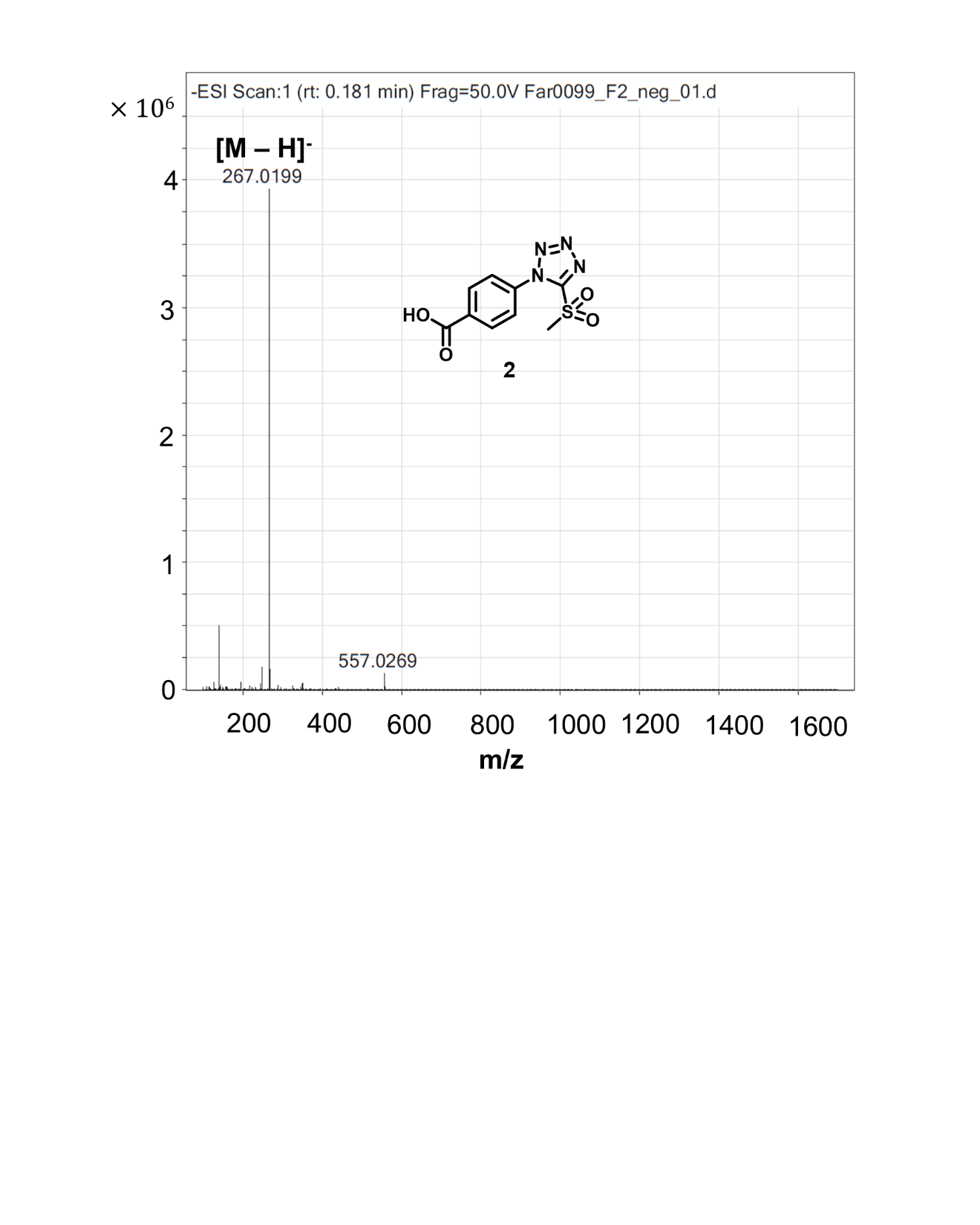


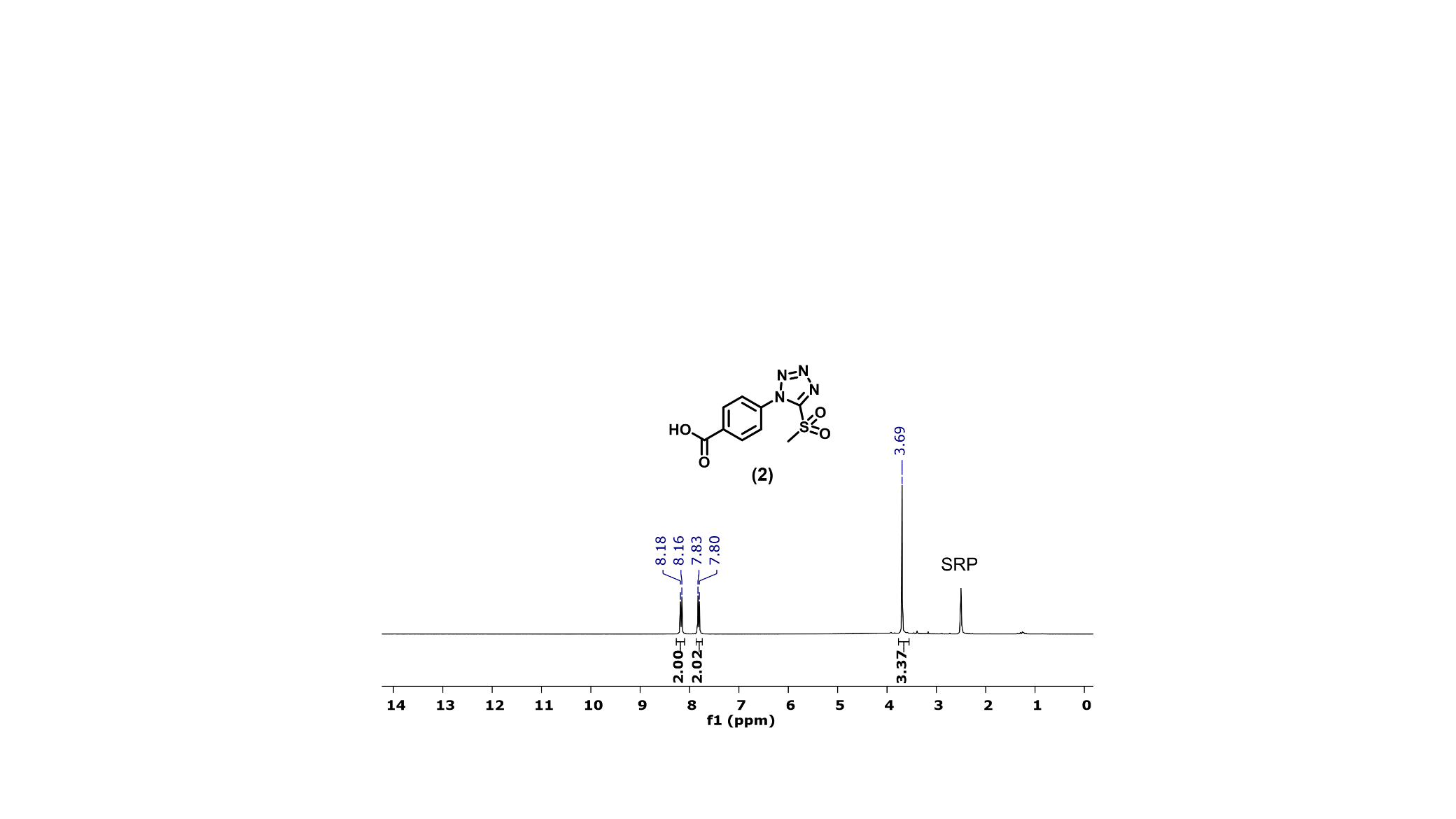


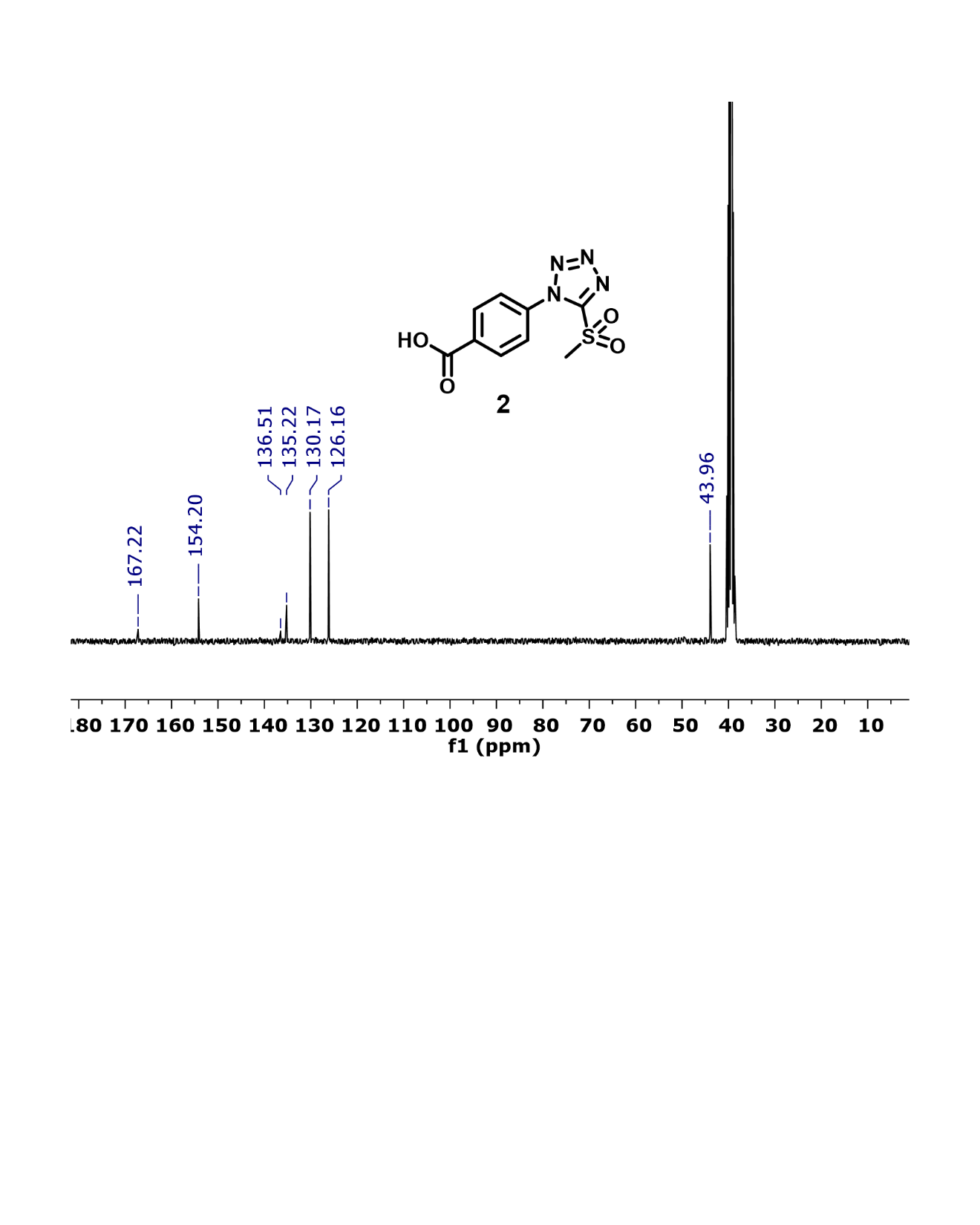


*1.2.3. Synthesis of compound perfluorophenyl 4-(5-(methylsulfonyl)-1H-tetrazol-1-yl)benzoate (3):*

Compound 3 was synthesized by dissolving 4-(5-(methylsulfonyl)-1H-tetrazol-1-yl)benzoic acid (0.5 g, 1.86 mmol, 1 equiv.), DMAP (0.022 g, 0.186 mmol, 0.1 equiv.) and pentafluorophenol (0.51 g, 2.79 mmol, 1.5 equiv.) in dry DCM under inert atmosphere. The solution was cooled to 0 °C and DCC (0.77 g, 3.72 mmol, 2 equiv.) was dissolved in dry DCM followed by addition to the above reaction mixture over a period of 5 minutes. The resulting mixture was stirred overnight at room temperature. The progress of reaction was monitored by TLC (DCM/MeOH 97:3 v/v). After the complete consumption of starting reactant, the organic solvent was filtered to remove the solid Dicyclohexylurea (DCU) by product and evaporated under vacuum. The crude product was purified by column chromatography by using DCM as eluent and the expected product was obtained as a white solid (0.546 g, 67.5 %).

ESI-MS+ (C_15_H_6_F_5_N_4_O_4_S): 433.0037 [M - H]^-^

^1^H NMR (300 MHz, Chloroform-*d*) δ (ppm) = 8.45 (d, *J* = 8.8 Hz, 2H, -CHAr), 7.96 (d, *J* = 8.7 Hz, 2H, -CHAr), 3.70 (s, 3H, -SO_2_Me).

^13^C NMR (75 MHz, Chloroform-*d*) δ (ppm) = 161.18, 154.30, 137.71, 132.44 (2C), 129.91, 125.45 (2C), 44.07.


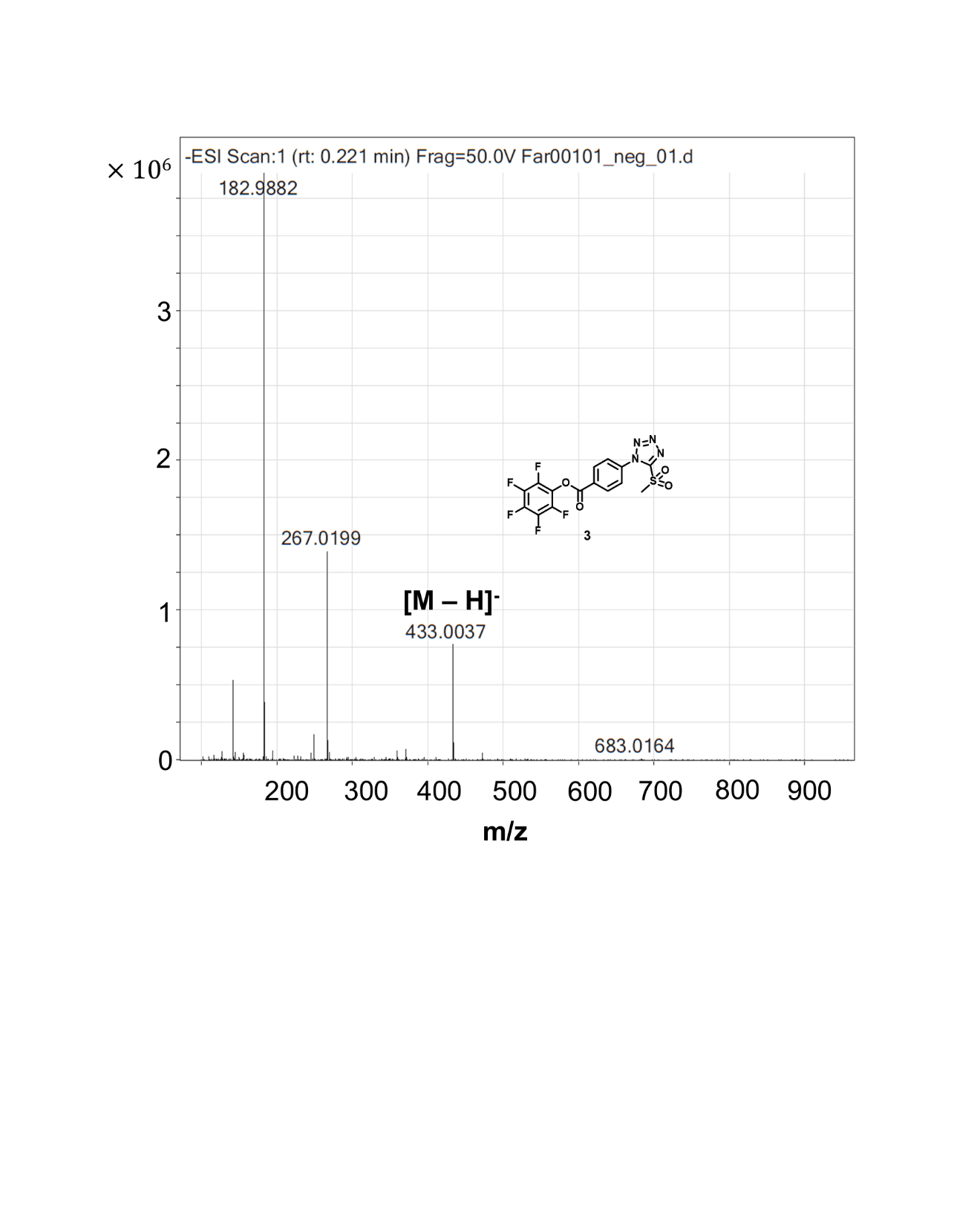


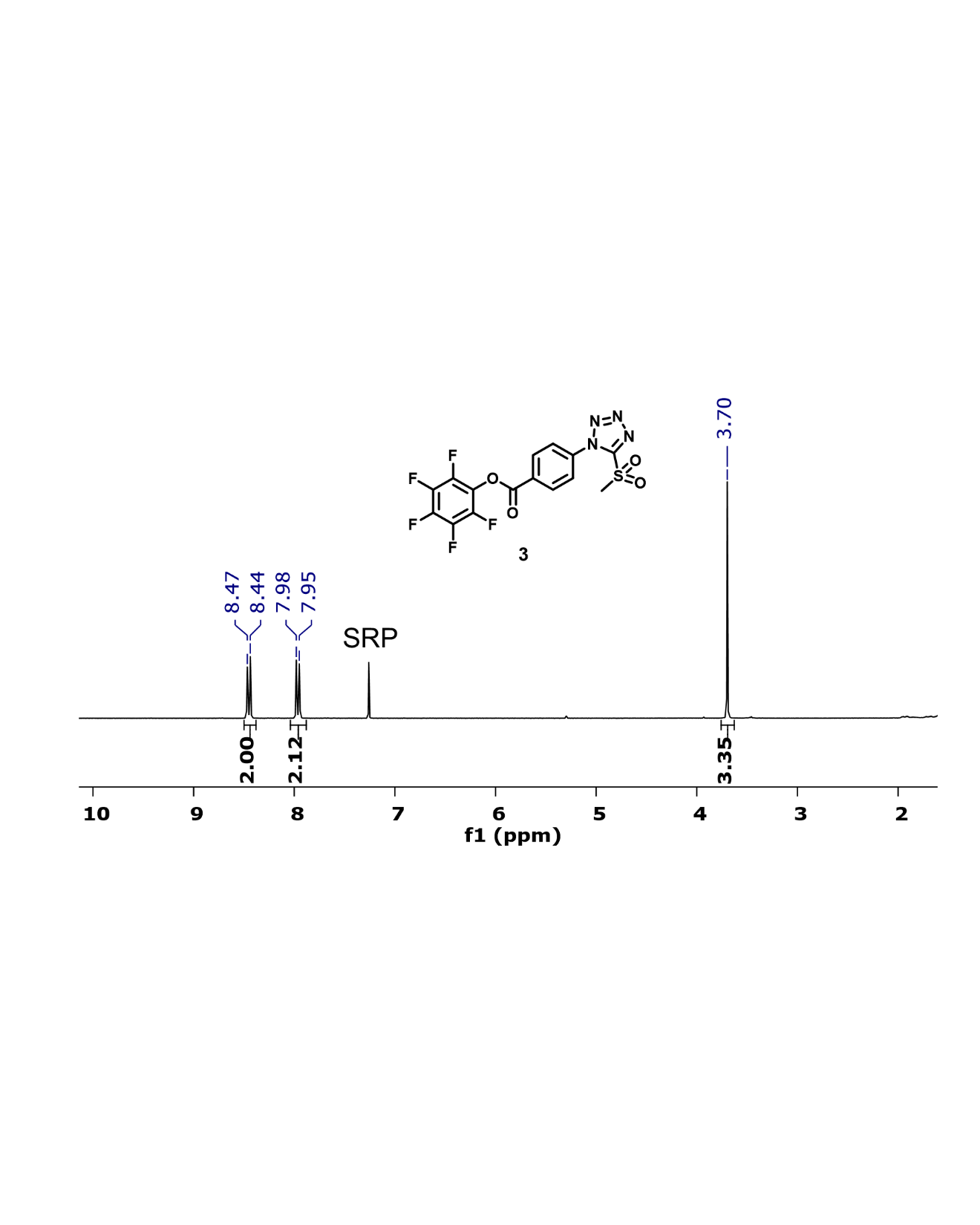


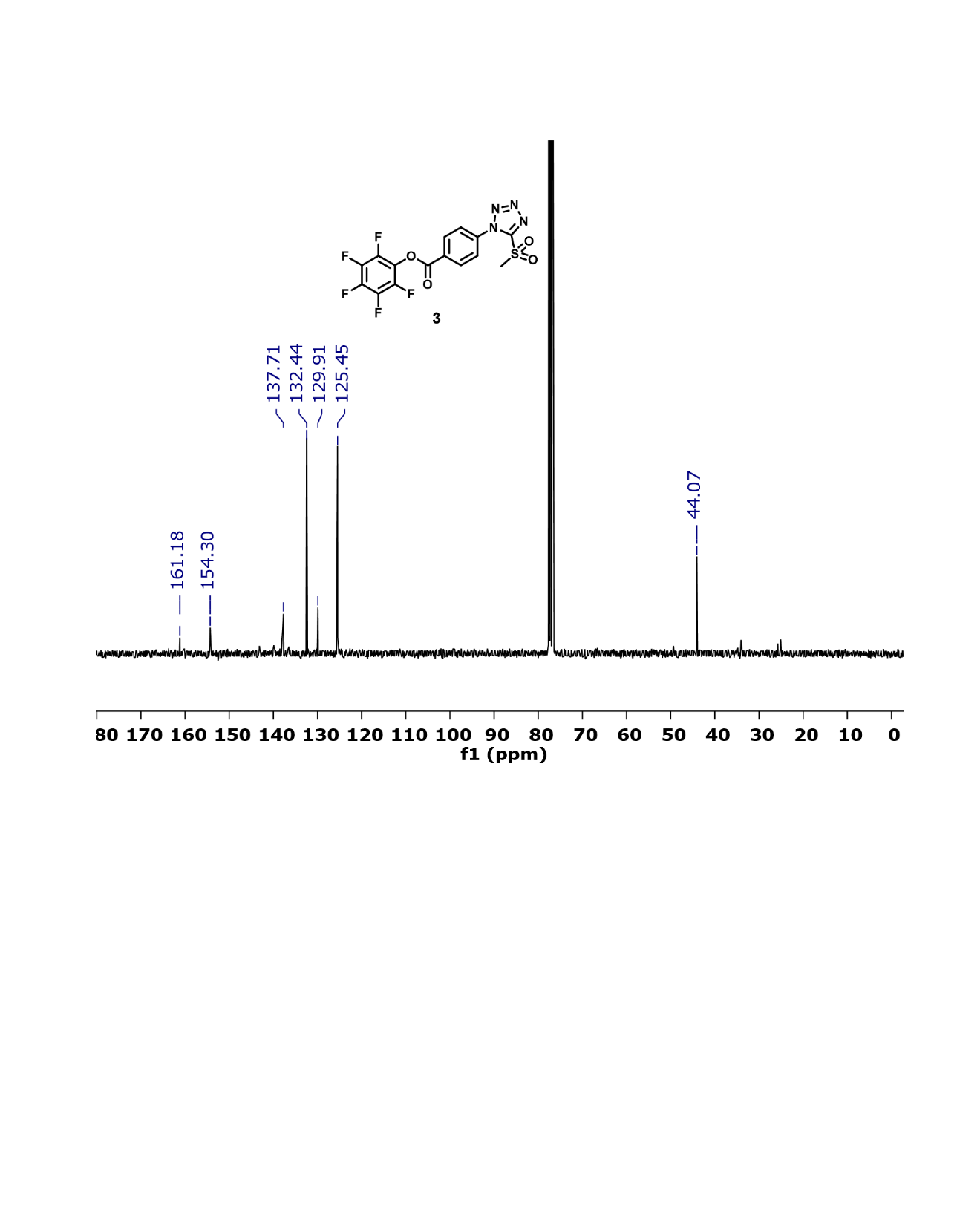


*1.2.4. Synthesis of PEG-4CONH-TzMS, 4* :

The PEG4-CONH-TzMS was synthesized by dissolving 20 kDa 4arm PEG-NH_2_.HCl (0.4 g, 0.02 mmol, 1 equiv.) and DIPEA (74.4 µL, 0.4 mmol, 20 equiv.) in 2 mL dry DMF under inert conditions and mixture was stirred at room temperature for 5 minutes. Pentafluorophenyl 4-(5-(methylsulfonyl)-1H-tetrazol-1-yl)benzoate (86.9 mg, 0.2 mmol, 10 equiv.) was dissolved in dry DMF (2 mL) and added to the above solution. The resulting solution was stirred at room temperature under inert atmosphere overnight. After 24 h the crude product was dialyzed with acetone and water and freeze dried to get the final compound as white solid in high yield (0.378 g, 92.5%). The degree of functionalization of > 95% was calculated from ^1^H-NMR spectra by end group determination using previously reported method.^2^ To calculate this, the signal from PEG back bone (3.97-3.37 ppm) was set to 493 H and the corresponding integral to bonded molecule was compared (aromatic protons 8.5 - 7.5 ppm).

^1^H NMR (300 MHz, Deuterium Oxide) δ (ppm) 8.08 (d, *J* = 8.6 Hz, 2H, -CHAr), 7.88 (d, *J* = 8.6 Hz, 2H, -CHAr), 3.96 (dd, *J* = 5.7, 3.3 Hz, 2H, -CH_2_NH), 3.87 – 3.30 (m, 489H, PEG core, and –SO_2_Me group).


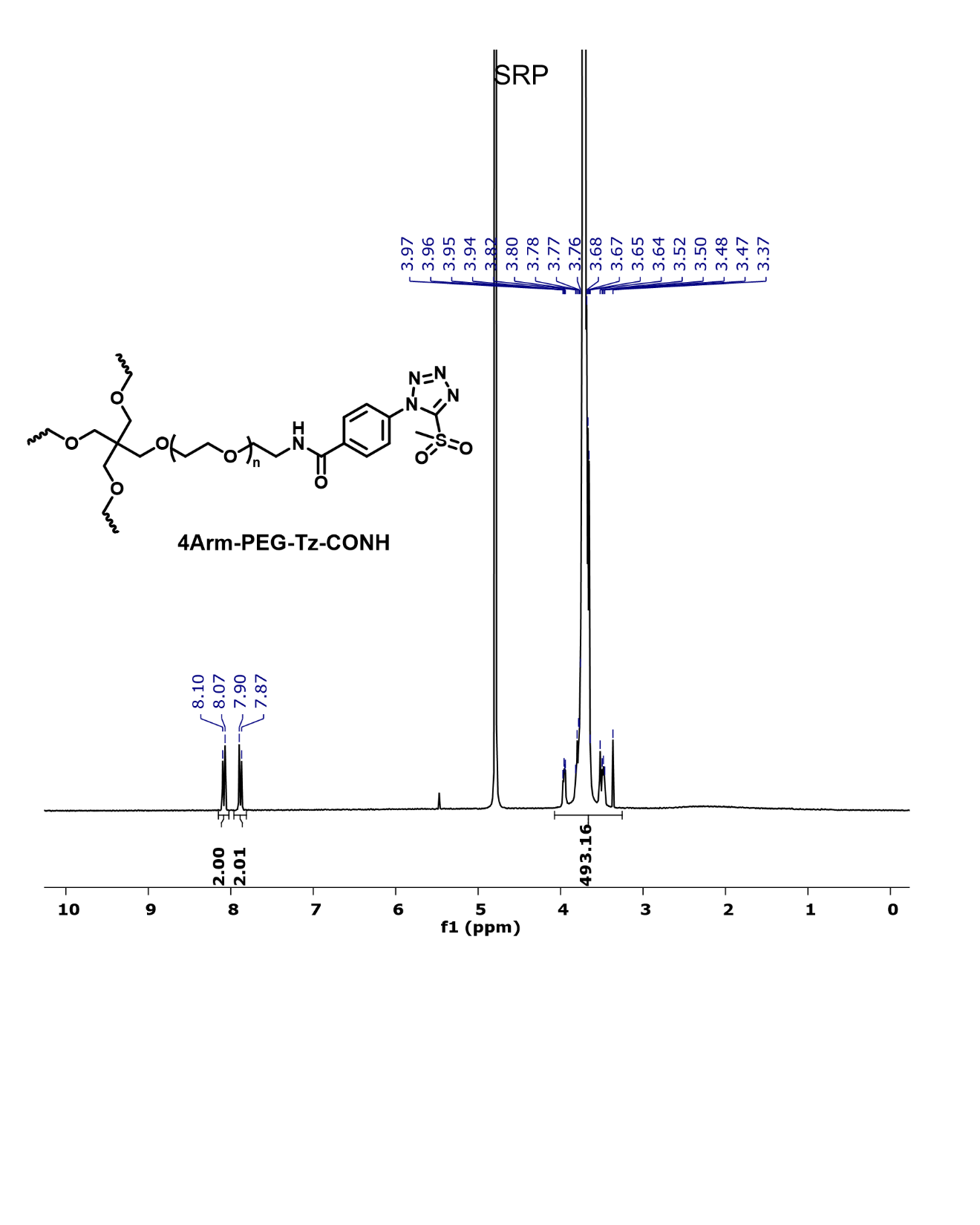


*Shelf life of compound (****3****):*

The shelf life of key intermediate (**3**) for PEG4-CONH-TzMS was analyzed by performing ^1^H-NMR in chloroform*-d* on day 1 of synthesis and after six months storage at 4 °C in dry conditions.


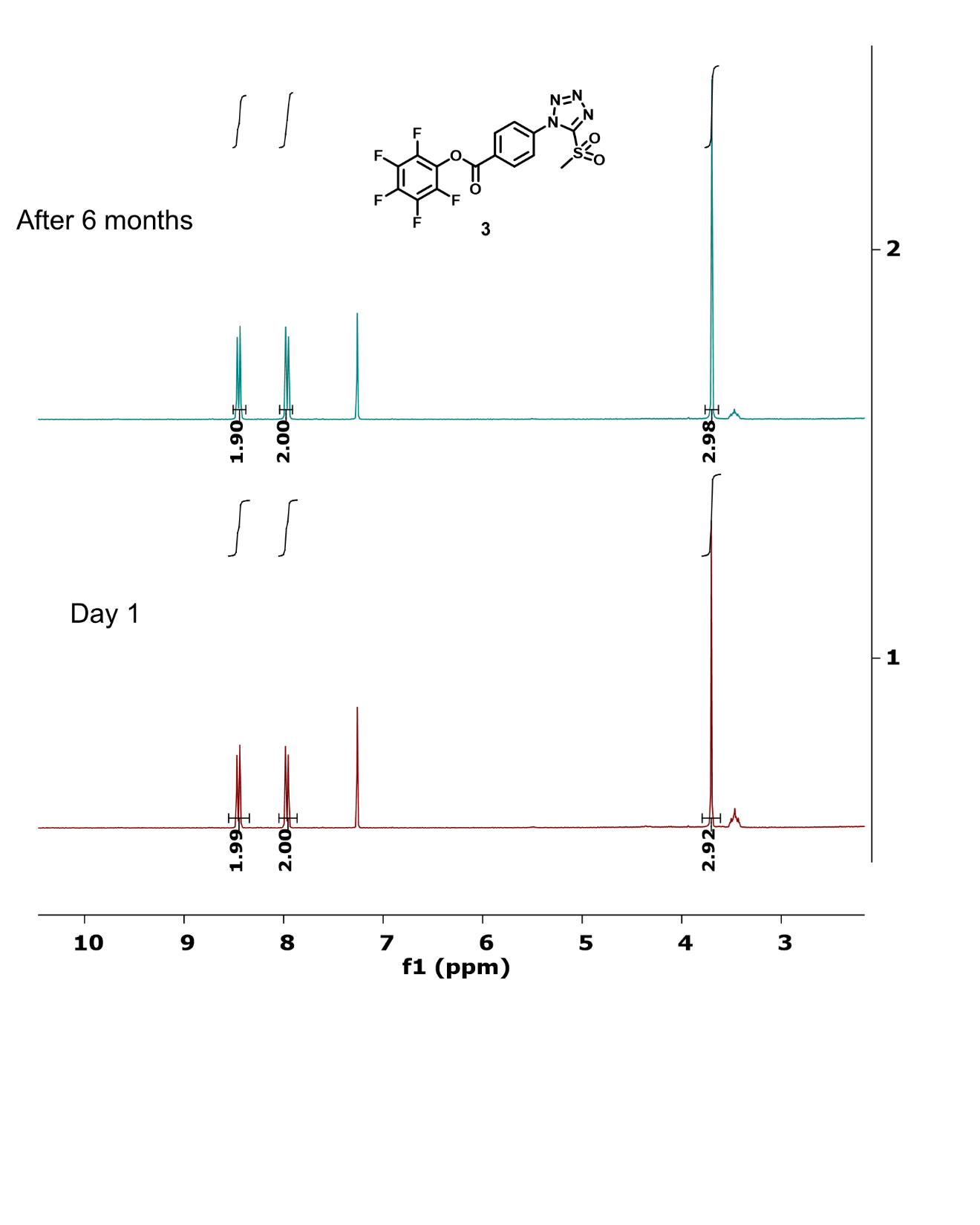


**Figure S1:** Stability of intermediate compound (3) followed by ^1^H-NMR in chloroform-d at day 1 and after 6 months of storage in dry state at 4 °C. No changes in NMR signals or decomposition were observed.

*Stability of PEG4-CONH-TzMS* :

The stability studies of PEG4-CONH-TzMS was performed by NMR in deuterated-PBS, following previously established protocol.^3^ The deuterated*-*PBS was prepared by dissolving 50 mg of salt tablet purchased commercially from Sigma Aldrich (Germany) into 5 mL of D_2_O. pH measurements of the resulting solution (pH 7.5) was recorded with a Eutech Elite pH Spear (Thermo Scientific) and used without further adjustment.^4^ The PEG4-CONH-TzMS (20 mg) was dissolved in 1 mL of deuterated-PBS buffer (pH 7.5) and NMR was recorded after regular intervals for up to 20 days. In between measurements, samples solutions were stored at 4°C.


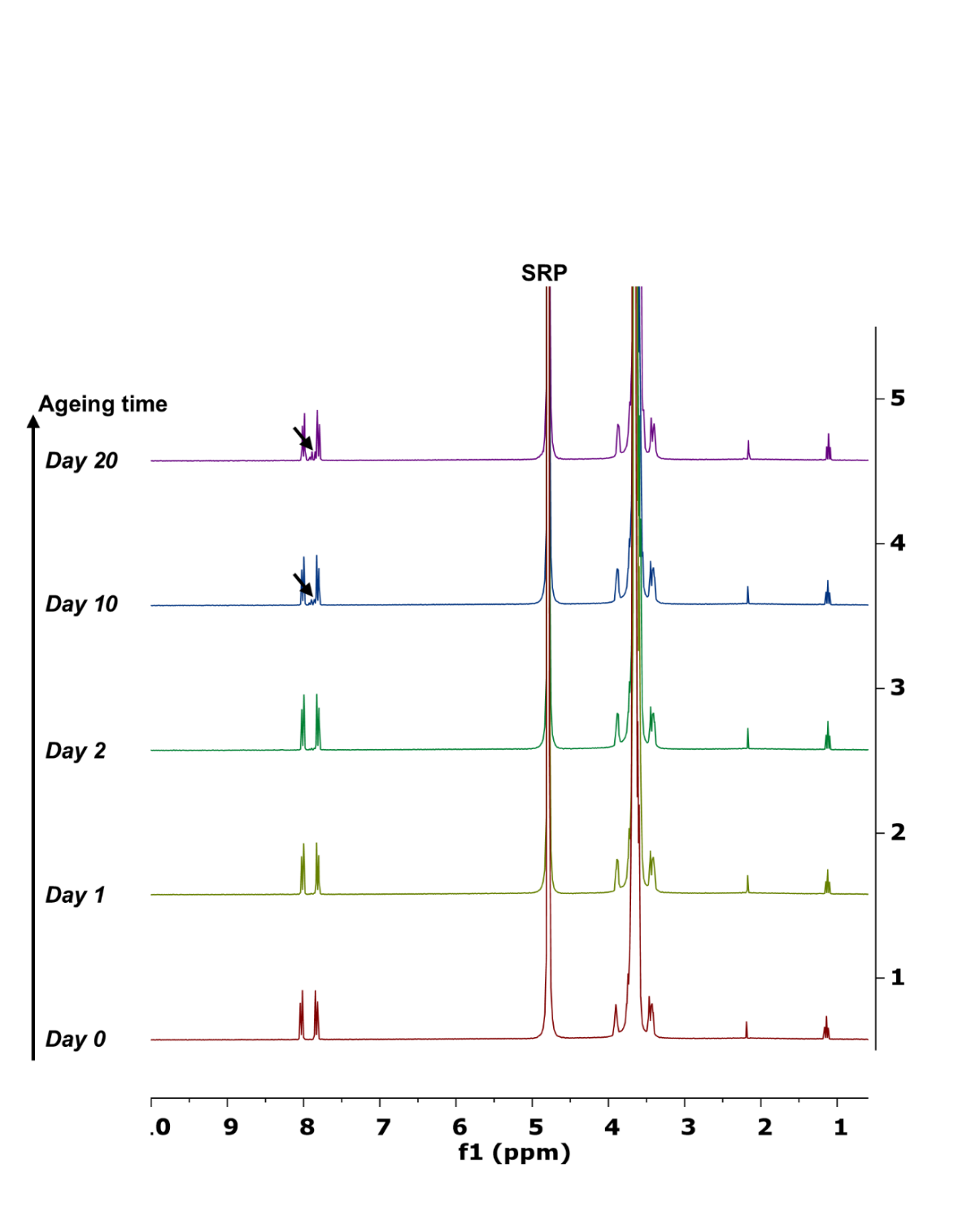


**Figure S2:** Stability of PEG4*-*CONH-TzMS followed by ^1^H-NMR in deuterated-PBS (pH 7.5) at increasing aging time of 0, 1, 2, 10 and 20 days. After 10 days a slight degradation in the aromatic region was observed indicated by black arrow. NMR analysis showed 94% compound was intact after 10 days and > 90% compound was intact after 20 days. The signal in NMR below 2.5 ppm are attributed to deuterated PBS.

*Reactivity of PEG4-CONH-TzMS*:

The solutions of TzMS (22.35 mg, 1 mM, 1 eq. ) and marcaptoethanol (0.14 µL, 2 mM ,2 eq.) in *d-*PBS (pH 7.5) were mixed in equal volume in NMR tube as described previously.^2^ The ^1^H-NMR of resulting mixture was recorded with 64 number of scans which take 10 minutes (minimum time to get good signal spectral resolution). The stability of resulting product was tested by measuring ^1^H-NMR of same solution at regular intervals for up to 6 days. After each measurement the NMR solution was stored at 4°C.


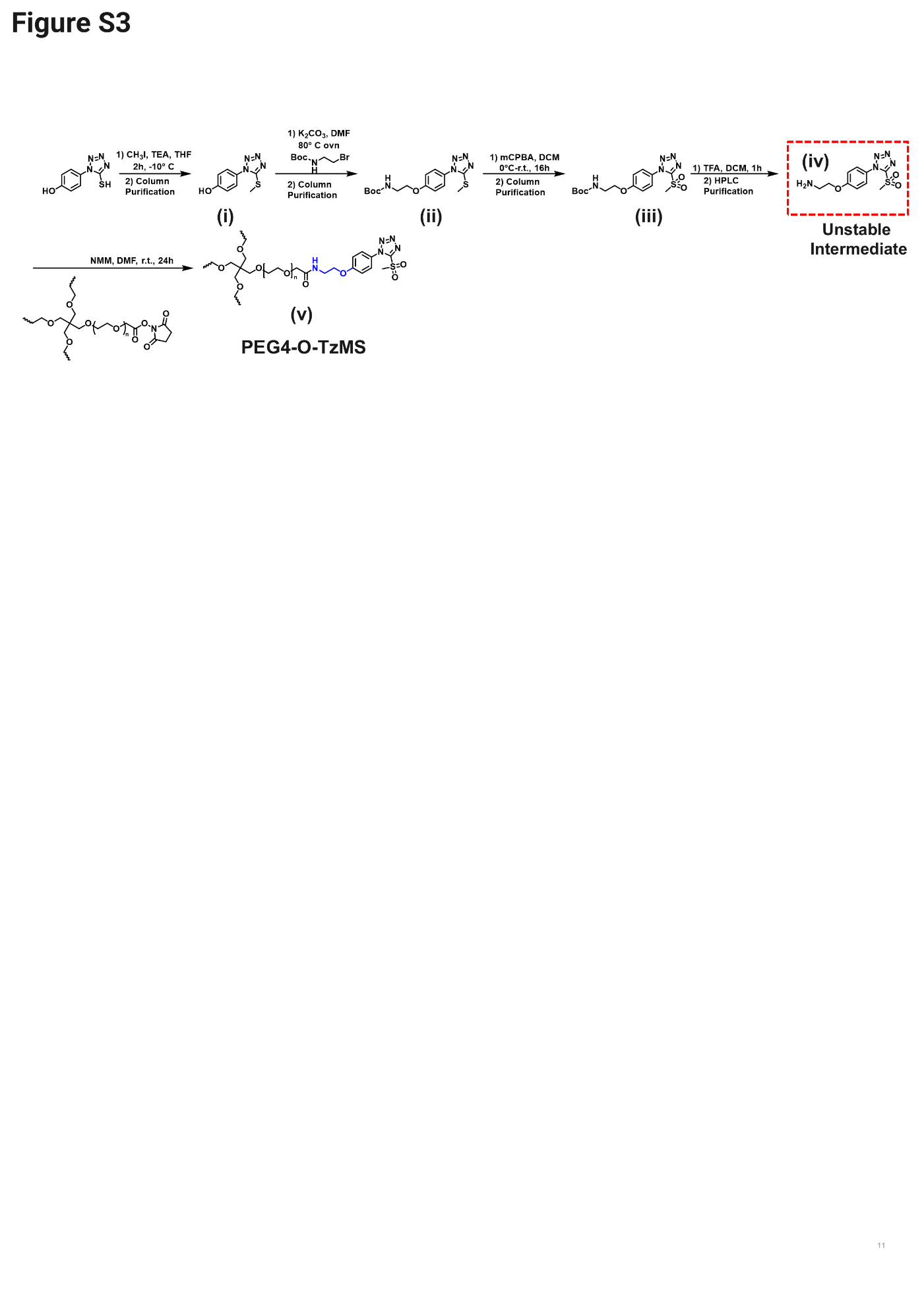


**Figure S3:** Synthesis scheme of PEG4-O-TzMS

## *Synthesis of TzMS derivatives with different polymeric back bones:*

Four TzMS derivatives were synthesized with different biocompatible polymeric backbones. For 8 arm-PEG-NH_2_ (40 kDa) and gelatin (type A, 300 bloom) the same reaction conditions were employed as described above for 4arm-PEG-NH_2_. ^5^ For polyvinyl alcohol (PVA, 31 kDa), the reaction was carried out in THF by using 1,5,7-Triazabicyclo[4.4.0]dec-5-ene (TBD) as base.^6, 7^ For heparin (15 kDa) derivatization a linker (ethylene diamine) was introduced to the compound (**c**) followed by using previously reported EDCI/NHS coupling.^8^ All the products were purified by dialysis and obtained in reasonable yields (65 - 85%).


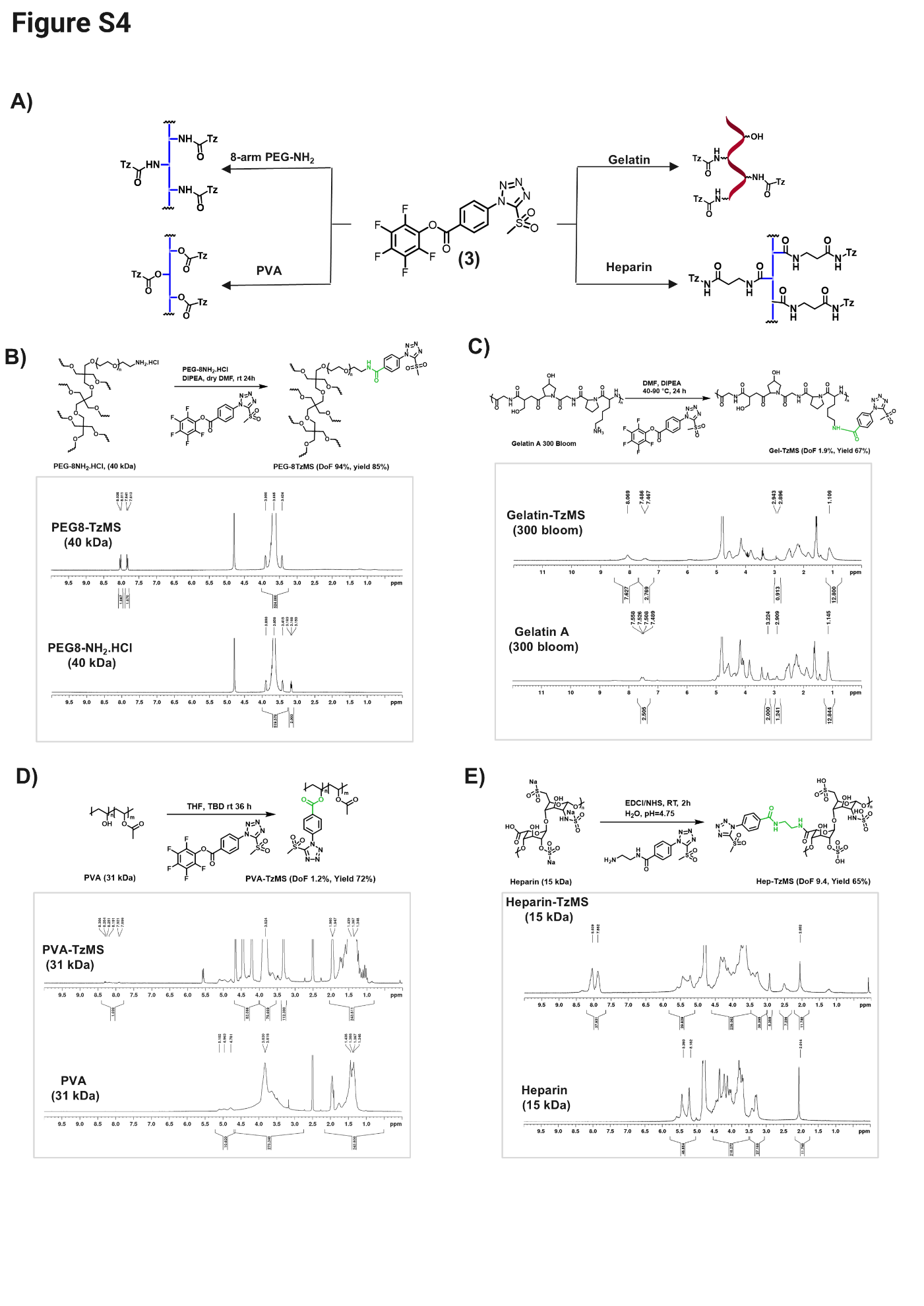


**Figure S4:** Synthesis of TzMS derivatives with different polymeric backbones. (**A**) Schematic illustration for synthesis of TzMS end-functionalized biocompatible polymers using reaction intermediate compound (**3**). (**B**) Synthesis scheme for 40 kDa 8 arm-PEG TzMS prepolymer. (**C**) Synthesis scheme for type A 300 bloom gelatin TzMS prepolymer. (**D**) Synthesis scheme for 31 kDa polyvinyl alcohol (PVA) TzMS prepolymer. (**E**) Synthesis scheme for 15 kDa heparin TzMS prepolymer.

**References**

1. Motiwala, H. F.; Kuo, Y.-H.; Stinger, B. L.; Palfey, B. A.; Martin, B. R., Tunable heteroaromatic sulfones enhance in-cell cysteine profiling. *Journal of the American Chemical Society* **2019,** *142* (4), 1801-1810.

2. de Miguel‐Jiménez, A.; Ebeling, B.; Paez, J. I.; Fink‐Straube, C.; Pearson, S.; Del Campo, A., Gelation Kinetics and Mechanical Properties of Thiol‐Tetrazole Methylsulfone Hydrogels Designed for Cell Encapsulation. *Macromolecular bioscience* **2023,** *23* (2), 2200419.

3. Paez, J. I.; de Miguel-Jiménez, A.; Valbuena-Mendoza, R.; Rathore, A.; Jin, M.; Glaser, A.; Pearson, S.; Del Campo, A., Thiol-methylsulfone-based hydrogels for cell encapsulation: reactivity optimization of aryl-methylsulfone substrate for fine-tunable gelation rate and improved stability. *Biomacromolecules* **2021,** *22* (7), 2874-2886.

4. Covington, A. K.; Paabo, M.; Robinson, R. A.; Bates, R. G., Use of the glass electrode in deuterium oxide and the relation between the standardized pD (paD) scale and the operational pH in heavy water. *Analytical Chemistry* **1968,** *40* (4), 700-706.

5. Asim, S.; Tabish, T. A.; Liaqat, U.; Ozbolat, I. T.; Rizwan, M., Advances in gelatin bioinks to optimize bioprinted cell functions. *Advanced healthcare materials* **2023,** *12* (17), 2203148.

6. Rémy, M.; Nierengarten, I.; Park, B.; Holler, M.; Hahn, U.; Nierengarten, J. F., Pentafluorophenyl esters as exchangeable stoppers for the construction of photoactive [2] rotaxanes. *Chemistry–A European Journal* **2021,** *27* (33), 8492-8499.

7. Anindita, D.; Patrick, T., Multifaceted Synthetic Route to Functional Polyacrylates by Transesterification of Poly (pentafluorophenyl acrylates). **2015**.

8. Hahn, D.; Sonntag, J. M.; Lück, S.; Maitz, M. F.; Freudenberg, U.; Jordan, R.; Werner, C., Poly (2‐alkyl‐2‐oxazoline)‐Heparin Hydrogels—Expanding the Physicochemical Parameter Space of Biohybrid Materials. *Advanced Healthcare Materials* **2021,** *10* (22), 2101327.
